# Renewable fatty acid ester production in *Clostridium*

**DOI:** 10.1038/s41467-021-24038-3

**Published:** 2021-07-16

**Authors:** Jun Feng, Jie Zhang, Yuechao Ma, Yiming Feng, Shangjun Wang, Na Guo, Haijiao Wang, Pixiang Wang, Pablo Jiménez-Bonilla, Yanyan Gu, Junping Zhou, Zhong-Tian Zhang, Mingfeng Cao, Di Jiang, Shuning Wang, Xian-Wei Liu, Zengyi Shao, Ilya Borovok, Haibo Huang, Yi Wang

**Affiliations:** 1 Department of Biosystems Engineering, Auburn University, Auburn, AL USA; 2 Center for Bioenergy and Bioproducts, Auburn University, Auburn, AL USA; 3 Department of Food Science and Technology, Virginia Tech, Blacksburg, VA USA; 4 School of Chemistry, National University (UNA), Heredia, Costa Rica; 5 Department of Chemical and Biological Engineering, Iowa State University, Ames, IA USA; 6 NSF Engineering Research Center for Biorenewable Chemicals, Iowa State University, Ames, IA USA; 7 Department of Applied Chemistry, University of Science and Technology of China, Hefei, China; 8 State Key Laboratory of Microbial Technology, Microbial Technology Institute, Shandong University, Qingdao, China; 9 The Shmunis School of Biomedicine and Cancer Research, Faculty of Life Sciences, Tel Aviv University, Ramat Aviv, Tel Aviv, Israel

**Keywords:** Metabolic engineering, Metabolic engineering, Applied microbiology

## Abstract

Bioproduction of renewable chemicals is considered as an urgent solution for fossil energy crisis. However, despite tremendous efforts, it is still challenging to generate microbial strains that can produce target biochemical to high levels. Here, we report an example of biosynthesis of high-value and easy-recoverable derivatives built upon natural microbial pathways, leading to improvement in bioproduction efficiency. By leveraging pathways in solventogenic clostridia for co-producing acyl-CoAs, acids and alcohols as precursors, through rational screening for host strains and enzymes, systematic metabolic engineering-including elimination of putative prophages, we develop strains that can produce 20.3 g/L butyl acetate and 1.6 g/L butyl butyrate. Techno-economic analysis results suggest the economic competitiveness of our developed bioprocess. Our principles of selecting the most appropriate host for specific bioproduction and engineering microbial chassis to produce high-value and easy-separable end products may be applicable to other bioprocesses.

## Abstract (web summary)

Esters can be used as fuels and specialty chemicals for food flavoring, cosmetic and pharmaceutical industries. Here, the authors systematically engineer clostridia, including discovery and deletion of prophages to increase strain stability, for the production of butyl acetate and butyl butyrate from corn stover at low cost.

## Introduction

Although tremendous efforts have been invested for biofuel and biochemical research, it is still challenging to generate robust microbial strains that can produce target products at desirable levels^1^. We hypothesize that, the production of high-value bioproducts which can be easily recovered from fermentation might be a solution to tackle the bottleneck in bioproduction. Fatty acid esters, or mono-alkyl esters, can be used as valuable fuels such as diesel components or specialty chemicals for food flavoring, cosmetic and pharmaceutical industries^2,3^. It is projected that the US market demand for fatty acid esters could reach $4.99 billion by 2025^4^. In addition, esters, with fatty acid and alcohol moieties, are generally hydrophobic and can be easily separated from fermentation, leading to high bioproduction efficiency.

Conventionally, esters are produced through Fischer esterification which involves high temperature and inorganic catalysts^5,6^. The reaction consumes a large amount of energy and generates tremendous wastes, and thus is not environmentally friendly^5^. On the other hand, ester production through biological routes is becoming more and more attractive because it is renewable and environmentally benign^3^. There are two primary biological pathways for ester production: one is through esterification of fatty acid and alcohol catalyzed by lipases^3,7^, and the other is based on condensation of acyl-CoA and alcohol catalyzed by alcohol acyl transferases (AATs)^5^. Previously, lipases from bacteria or fungi have been employed for catalyzing esterification for ester production^8^. For instance, lipase from *Candida sp*. has been recruited to drive production of butyl butyrate (BB) with *Clostridium tyrobutyricum*. *C. tyrobutyricum*, a natural hyper-butyrate producer, could generate 34.7 g/L BB with supplementation of lipase and butanol^9^. However, in such a process, the supplemented enzyme accounts for a big cost and meanwhile the operation needs to be carefully managed to achieve the optimum performance of esterification^9^. Therefore, a whole microbial cell factory capable of ester production in one pot is highly desired. Some microorganisms can naturally produce esters^3^. For instance, *Kluyveromyces marxianus* DSM 5422 has been reported to produce 8.92 g/L of ethyl acetate (EA) in the fermentation^10^. *Saccharomyces cerevisiae* could also produce various esters with its native AATs, but generally at very low levels (<1 g/L)^11^. Metabolic engineering of microorganisms for ester production by introducing the heterologous ester-producing pathways has been attempted by various researchers. Horton et al. introduced the AAT genes of *S. cerevisiae* into *C. acetobutylicum* and *Escherichia coli* and obtained 0.6 mM and 1.8 mM isoamyl acetate, respectively^12^. The same group of researchers also achieved the production of small amount of EA, butyl acetate (BA) and BB when they introduced AAT genes from *S. cerevisiae* and strawberry into *E. coli*^13^. Bohnenkamp et al. engineered *E. coli* to produce up to 3.8 g/L EA by overexpressing the *eat1* gene from *Wickerhamomyces anomalus*^14^. Rodriguez et al. metabolically engineered *E. coli* to produce esters by introducing heterologous AATs^5^. Although the production of some acetate esters can reach decent levels (such as 17.2 g/L isobutyl acetate), the production of most of the esters was rather low, probably due to the unavailability of intrinsic substrates/precursors and limited tolerance of *E. coli* to organic endproducts.

In this work, we report highly efficient fatty acid ester production to high levels using engineered clostridia. We select solventogenic clostridia to take advantage of their natural pathways for co-producing acyl-CoAs (acetyl-CoA and butyryl-CoA), fatty acids (acetate and butyrate), and alcohols (ethanol and butanol), either as intermediates or endproducts; in addition, solventogenic clostridia tend to possess good tolerance to toxic endproducts thanks to their intrinsic capacity for high-level solvent production. We hypothesize that clostridia can be excellent microbial platforms to be engineered for efficient ester production by introducing heterologous AATs and/or lipase genes. Indeed, through rational screening for host strains (from four well-known clostridial species) and enzymes (AATs and lipase), systematic metabolic engineering—including rational organization of ester-synthesizing enzymes inside of the cell, and elimination of the prophage-like regions, we ultimately obtain two strains which can produce 20.3 g/L BA and 1.6 g/L BB in extractive batch fermentations.

## Results

### Screening of host strains and genes for ester production

**Fig. 1:**
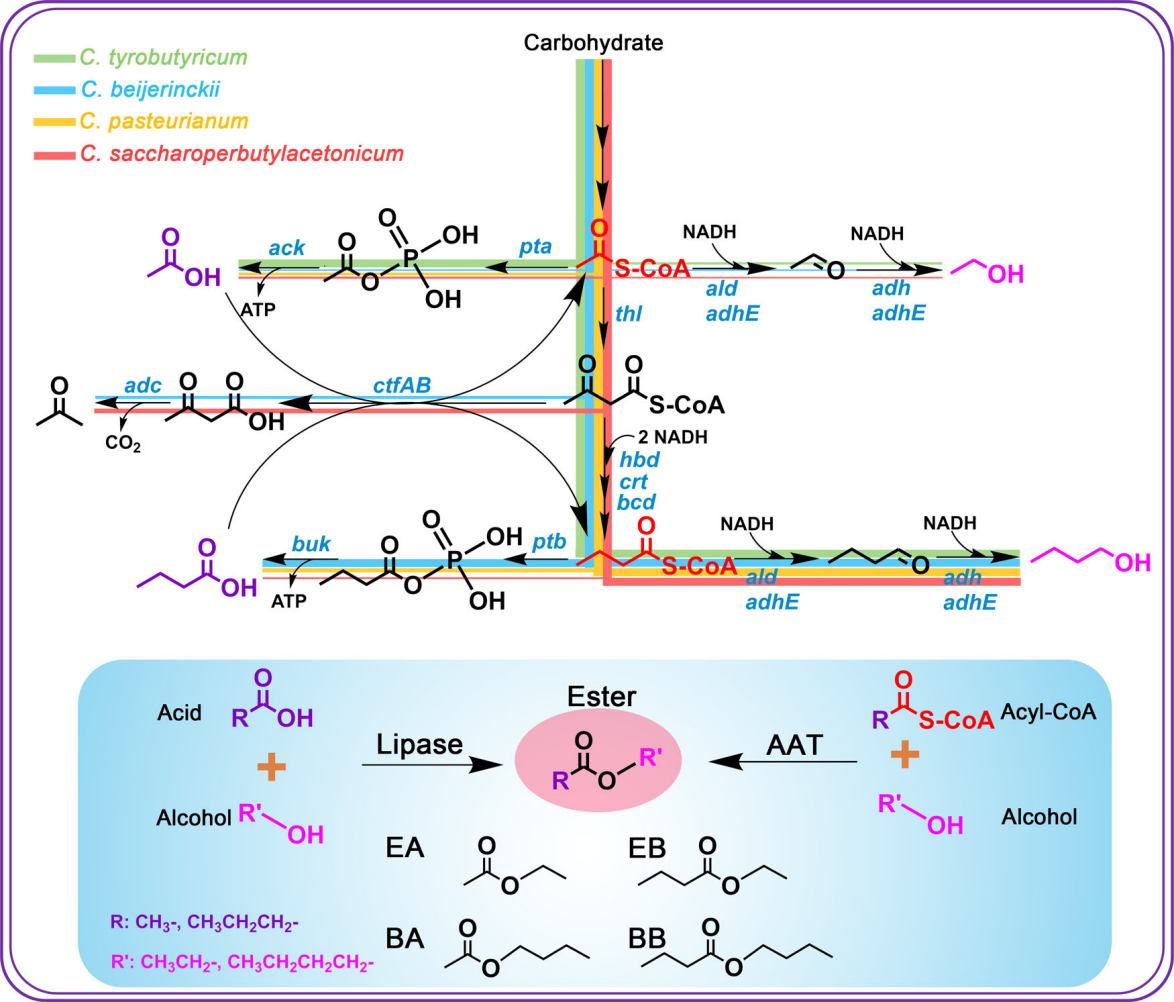
Engineering of solventogenic clostridia for fatty acid ester production. Top: Five strains out of four representative clostridial species were selected and evaluated as the host to be engineered for ester production in this study. We hypothesized that the different metabolic fluxes within different strains would make a big difference for the desirable ester production. The metabolic pathways of the four different species were represented in four different colors. Bottom: Fatty acid esters could be synthesized through two primary biological pathways: one is through the esterification of fatty acid and alcohol catalyzed by lipases, and the other is through the condensation of acyl-CoA and alcohol catalyzed by alcohol acyl transferases (AATs). Key genes in the pathway: *pta* phosphotransacetylase, ack acetate kinase, *thl* thiolase, *hbd* beta-hydroxybutyryl-CoA dehydrogenase, *crt* crotonase, *bcd* butyryl-CoA dehydrogenase, *adh* alcohol dehydrogenase, *adhE* aldehyde-alcohol dehydrogenase, *adc* acetoacetate decarboxylase, *ctfAB* CoA transferase, *ptb* phosphotransbutyrylase, *buk* butyrate kinase*, ald* aldehyde dehydrogenase. EA: ethyl acetate, EB: ethyl butyrate, BA: butyl acetate, BB: butyl butyrate.

We considered clostridia as ideal platforms for ester production thanks to their intrinsic intermediates (fatty acids, acyl-CoAs, and alcohols) serving as precursors for ester biosynthesis (Fig. 1). We hypothesized that different flux levels of these precursors within various clostridial strains would make a big difference for the specific type(s) of ester production. Therefore, we selected five strains (from four representative species) including *C. tyrobutyricum* ∆*cat1::adhE1*^15^*, C. tyrobutyricum* ∆*cat1::adhE2*^15^, *C. pasteurianum* SD-1^16^, *C. saccharoperbutylacetonicum* N1-4-C^17^ and *C. beijerinckii* NCIMB 8052^18^ to evaluate their capabilities for ester production through metabolic engineering. We included both *C. tyrobutyricum* ∆*cat1::adhE1* and ∆*cat1::adhE2* here because they produce different levels (and thus ratios) of butanol and ethanol^15,19^. Esters can be synthesized either through esterification of acid and alcohol catalyzed by lipase, or through condensation of acyl-CoA and alcohol catalyzed by AATs (Fig. 1). Previously, lipase B (CALB) from *Candida antarctica* has been employed for efficient ester production through esterification^9,20^. In addition, four AATs including VAAT^23^, SAAT^21,23^, ATF1^2,11^, EHT1^22^ have been recruited for ester production in various hosts^5,23–25^. Therefore, here, we evaluated all these genes in our clostridial hosts for ester production.

**Fig. 2:**
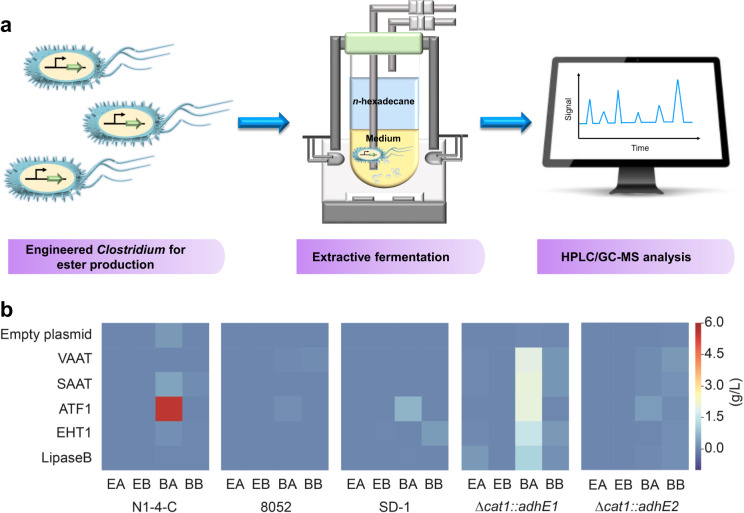
Screening of strains and enzymes for ester synthesis. **a** Schematic representation of procedures for extractive ester fermentation. **b** Heatmap results showed the ester production in various *Clostridium* strains with the overexpression of different enzymes. Empty plasmid: expression of pMTL82151 (for *C. saccharoperbutylacetonicum* N1-4-C, *C. pasteurianum* SD-1, *C. tyrobutyricum cat1::adhE1* and *cat1::adhE2*) or pTJ1 (for *C. beijerinckii* 8052) as the control; EA: ethyl acetate, EB: ethyl butyrate, BA: butyl acetate, BB: butyl butyrate. Source data underlying Fig. 2b are provided as a Source Data file.

Six plasmids (pMTL-P_*cat*_-*vaat*, pMTL-P_*cat*_-*saat*, pMTL-P_*cat*_-*atf1*, pMTL-P_*cat*_-*eht1*, pMTL-P_*cat*_-*lipaseB* as well as pMTL82151 as the control) were individually transformed into *C. saccharoperbutylacetonicum* N1-4-C, *C. pasteurianum* SD-1, *C. tyrobutyricum* Δ*cat1::adhE1* and Δ*cat1::adhE2*, respectively. While pTJ1-P_*cat*_-*vaat*, pTJ1-P_*cat*_-*saat*, pTJ1-P_*cat*_-*atf1*, pTJ1-P_*cat*_-*ehtl* and pTJ1-P_*cat*_-*lipaseB* as well as pTJ1 were transformed into *C. beijerinckii* 8052. Fermentations were performed (Fig. 2a), and results were shown in Fig. 2b. Four types of esters were detected: EA, BA, ethyl butyrate (EB) and BB. Interestingly, control strains with the empty plasmid (pMTL82151 or pTJ1) also produced noticeable EA, BA and BB. This could be because: (1) the endogenous lipase in clostridia can catalyze ester production^17^; (2) *catP* on pMTL82151 encoding a chloramphenicol acetyltransferase (belonging to the same class of enzymes as AATs) has AAT activities^5,26^.

Based on the results, it could be concluded that ATF1 is more favorable for BA production. All strains with *atf1* produced higher levels of BA compared to the same strain but with the overexpression of other genes (Fig. 2b). While VAAT, SAAT and EHT1 seemed to have better activities for BB production. Among all the strains, *C. saccharoperbutylacetonicum* FJ-004 produced the titer of 5.5 g/L BA. Previously, *C. acetobutylicum* CaSAAT (with the overexpression of *saat* from *Fragaria xananassa*) was reported to produce 8.37 mg/L BA^25^. While this manuscript was prepared, a newly engineered *C. diolis* strain was reported to produce 1.37 g/L BA^27^. Here, the BB production of 0.3 g/L was observed in *C. pasteurianum* J-5 with the overexpression of *eht1*, which is higher than the recently reported 50.07 mg/L in an engineered *C. acetobutylicum*^25^. Six of our engineered strains could also produce small amount of EB with the titer up to 0.02 g/L been observed in *C. pasteurianum* J-5. With the *lipaseB* overexpression, *C. tyrobutyricum* JZ-6 could generate 0.3 g/L EA. This was significantly higher than other strains tested in this work (mostly < 0.01 g/L). As we reported previously, the mother strain *C. tyrobutyricum* Δ*cat1::adhE1* could produce 20.8 g/L acetate and 5.3 g/L ethanol (precursors for EA synthesis) during a batch fermentation, which might have enabled high-level EA production in *C. tyrobutyricum* JZ-6^15^.

The production levels of BA and BB achieved above are both significantly higher than the previously reported in microbial hosts. In comparison, the BA level is much higher than BB level, and thus has greater potentials toward economic viability. Therefore, in the following steps, we primarily focused on systematic metabolic engineering of the strain for further enhanced BA production.

### Enhancement of NADH availability

Based on the pathway, for either butanol (the precursor for BA production) or BA production, a large amount of reducing power (NADH) is required. Therefore, we set out to increase the availability of NADH to improve BA production. It has been reported that in the solventogenic *C. acetobutylicum* cells, the NADPH/NADP^+^ ratio is at least 70 times higher than the NADH/NAD^+^ ratio^28,29^. It therefore indicates in a sense that there is surplus NADPH available that can be leveraged for enhanced production of reduced endproducts. Indeed, by overexpressing a gene encoding a mutated aldehyde/alcohol dehydrogenase that possesses increased affinity for NADPH, Jang et al. significantly improved the butanol production in an engineered *C. acetobutylicum* strain^30^. In another study, by replacing the NADH-dependent *hbd* (encoding beta-hydroxybutyryl-CoA dehydrogenase) in *C. acetobutylicum* with the strictly NADPH-dependent *hbd1* from *C. kluyveri*, butanol/ethanol ratio and butanol flux were improved by 6- and 1.6-fold, respectively; meanwhile, the butanol yield reached 0.34 g/g in a simple mineral media without an organic nitrogen source^28^.

On the other hand, the recently discovered flavin-based electron-bifurcating NADH-dependent reduced ferredoxin:NADP^+^ oxidoreductase NfnAB catalyzes the transhydrogenation reaction among NAD^+^, NADP^+^, and ferredoxin^31^. Electron-bifurcating Nfn widely exists in anaerobes such as solventogenic clostridia, and catalyzes the endergonic reduction of NADP^+^ with NADH coupled to the exergonic reduction of NADP^+^ with reduced ferredoxin under appropriate physiological conditions. The ferredoxin-dependent transhydrogenase plays an important role in maintaining the redox balance in vivo, and participates in various metabolisms including acetogenesis, butyrate formation, and H_2_ production^32^. In this study, we hypothesized that the gene cluster of *Cspa_c47560/Cspa_c47550* encodes an enzymatic complex possessing a function similar to NfnAB for NADH dehydrogenation and ferredoxin oxidation (see Supplementary Note 1, Supplementary Method 1), and, by deleting it (in full or partially), butanol (in butanol-producing mother strain) and BA (in BA-producing mutant) production would be boosted because of the potentially increased NADH availability (by decreasing the conversion of NADH to NADPH). Thus, we deleted *Cspa_c47560* in N1-4-C and generated FJ-100. Further, FJ-101 was constructed based on FJ-100 for BA production. Results demonstrated that, although butanol production in FJ-100 was only slightly improved (16.5 g/L vs. 15.8 g/L in N1-4-C; Supplementary Fig. 1), BA production in FJ-101 was remarkably enhanced compared to FJ-004 (7.8 g/L vs. 5.5 g/L). Based on our experiences, because *C. saccharoperbutylacetonicum* N1-4 (HMT) (or N1-4-C) mother strain can naturally produce very high level of butanol, it was generally very difficult to further improve butanol production in *C. saccharoperbutylacetonicum* through simple metabolic engineering strategies^33^. This is likely the case in FJ-100 (comparing to N1-4-C). However, the increased NADH availability with *Cspa_c47560* deletion in FJ-101 (see Supplementary Note 1 and Supplementary Fig. 2) would enable an enhanced instant flux/availability of butanol which would serve as the precursor for BA production and thus enhance BA production in FJ-101. In this sense, the total butanol generated during the process (including the fraction serving as the precursor for BA production and the other fraction as the endproduct) in FJ-101 would be actually much higher than in FJ-004.

### Tuning of acetyl-CoA availability/flux

**Fig. 3:**
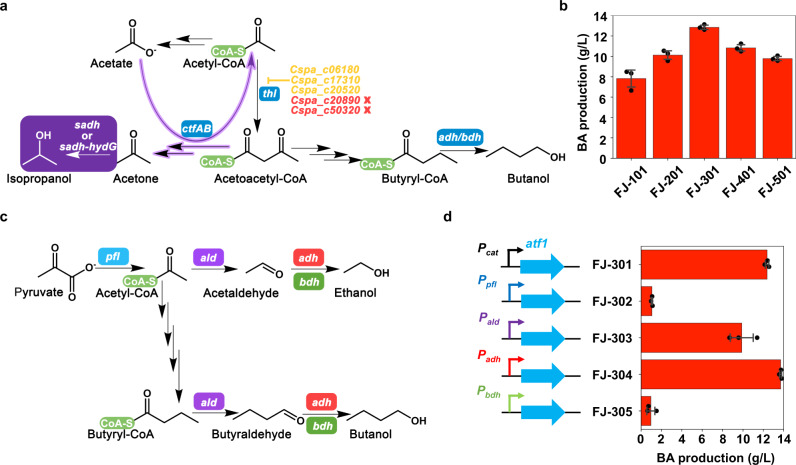
Enhancing butyl acetate production through increasing the availability of acetyl-CoA and dynamically expressing the *atf1* gene. **a** Introduction of isopropanol synthesis pathway (shaded in purple) and deletion of thiolase genes. The pathways in purple arrows represent the regeneration of acetyl-CoA. There are five annotated genes encoding thiolase in *C. saccharoperbutylacetonicum*, only two (in red) of which could be deleted (see Supplementary Note 2). **b** The fermentation results for butyl acetate (BA) production with various mutant strains corresponding to the genetic manipulations in (**a**). Data are presented as mean values ± SD (*n* = 3). **c** Four promoters associated with the biosynthesis of acetyl-CoA or alcohols in the pathway were selected to drive the expression of *atf1*. **d** The fermentation results for BA production with various mutant strains in which different promoters were used to drive the expression of *atf1* as illustrated in (**c**). Data are presented as mean values ± SD (*n* = 3). Key genes in the pathway: *adh* alcohol dehydrogenase, *bdh* butanol dehydrogenase, *ctfAB* CoA transferase, *sadh* secondary alcohol dehydrogenase, *hydG* putative electron transfer protein, *pfl* pyruvate formate lyase, *ald* aldehyde dehydrogenase. Source data underlying Fig. 3b and d are provided as a Source Data file.

Besides butanol, acetyl-CoA is the other primary precursor for BA production. We set out with two strategies to attune the acetyl-CoA availability/flux in attempt to further enhance BA production: (1) to decrease the flux of acetyl-CoA to acetoacetyl-CoA by deleting the thiolase gene, and (2) to pull the metabolic flux to acetone to enhance the acetate reassimilation (and thus the acetyl-CoA regeneration) by integrating the pathway of converting acetone into isopropanol (Fig. 3a). While we initially failed with (1) despite multiple trials (see Supplementary Note 2), we easily achieved (2) as we did previously when we engineered *C. saccharoperbutylacetonicum* for high level isopropanol-butanol-ethanol (IBE) production as also described below^34^.

The *sadh* gene in *C. beijerinckii* B-593 encoding a secondary alcohol dehydrogenase can convert acetone into isopropanol^35^. The *hydG* gene in the same operon as *sadh* encodes a putative electron transfer protein. It has been demonstrated to play important roles for the conversion of acetone into isopropanol^34,36^. In this study, either *sadh* alone or the *sadh*-*hydG* gene cluster was integrated into the chromosome of FJ-100, generating FJ-200 and FJ-300, respectively. Further, by introducing pMTL-cat-*atf1*, FJ-201 and FJ-301 were obtained. Compared to FJ-101, about 50% of the acetone could be converted into isopropanol in FJ-201, while ~95% of the acetone in FJ-301 could be converted into isopropanol. The total titers of acetone plus isopropanol in FJ-301 and FJ-201 were 6.6 g/L and 5.1 g/L respectively, both of which were higher than 4.8 g/L acetone in FJ-101. More significantly, BA production in FJ-201 and FJ-301 has been remarkably increased compared to FJ-101, and reached 10.1 g/L and 12.9 g/L, respectively (Fig. 3b). This result is somewhat surprising. One possible reason could be because isopropanol is less toxic than acetone to the cells; however, our cell growth tolerance experiments ruled out this possibility (Supplementary Note 2, Supplementary Fig. 3, and Supplementary Method 2). Another possible explanation is that, by pulling flux from acetone to isopropanol, the transferring of CoA from acetoacetyl-CoA to acetate driven by the CoA transferase would be boosted, resulting in increased instant availability of acetyl-CoA, and thus enhanced BA production (also see Supplementary Note 2). Anyway, it warrants a further investigation on this matter through, for example, a systematic metabolic flux analysis and/or metabolic modelling analysis.

### Dynamic expression of *atf1*

In our engineered strain, BA is synthesized through condensation of butanol and acetyl-CoA catalyzed by ATF1. The constitutively high expression of ATF1 would not necessarily lead to high BA production. For example, BA production in FJ-008 in which *atf1* was driven by the constitutive strong promoter P_*thl*_ from *C. tyrobutyricum* was actually much lower (3.5 g/L vs. 5.5 g/L) than in FJ-004 in which *atf1* was expressed under the promoter P_*cat*_ from *C. tyrobutyricum*. We hypothesized that, in order to obtain more efficient BA production, the synthesis of ATF1 should be dynamically controlled and thus synchronous with the synthesis of precursors (butanol or acetyl-CoA). Therefore, for the next step, we attempted to evaluate various native promoters for *atf1* expression, and identify the one(s) that can enable an appropriately dynamic expression of ATF1 and lead to enhanced BA production.

Four promoters associated with the synthesis of BA precursors were selected to drive the *atf1* expression, and four strains were constructed correspondingly for BA production (Fig. 3c). Fermentation results were shown in Fig. 3d. Indeed, distinct results for BA production were observed in these strains. The BA level was only 1.0 and 1.1 g/L in FJ-305 and FJ-302 with P_*bdh*_ and P_*pfl*_ for *atf1* expression, respectively. P_*ald*_ is an important promoter in *C. saccharoperbutylacetonicum*, which can sense the acidic state and switch cell metabolism from acidogenesis to solventogenesis^37^. FJ-303 with the *atf1* expression driven by P_*ald*_ produced 9.9 g/L BA, which was still 20% lower than in FJ-301. Interestingly, FJ-304 in which *atf1* was expressed under P_*adh*_ produced 13.7 g/L BA, which was about 10.5% higher than in FJ-301. Based on the results, we speculated that the promoter of *adh* (which encodes the key enzyme catalyzing butanol and ethanol production) might have resulted in the more appropriate dynamic expression of *atf1* in line with the flux of the precursors and thus led to enhanced BA production. On the other hand, ethanol production in FJ-304 was also lower than in FJ-301 (0.3 g/L vs. 0.6 g/L). The enhanced BA production in FJ-304 consumed more acetyl-CoA, and thus decreased production of ethanol, which also needed acetyl-CoA as the precursor.

### Rational organization of BA-synthesis enzymes

**Fig. 4:**
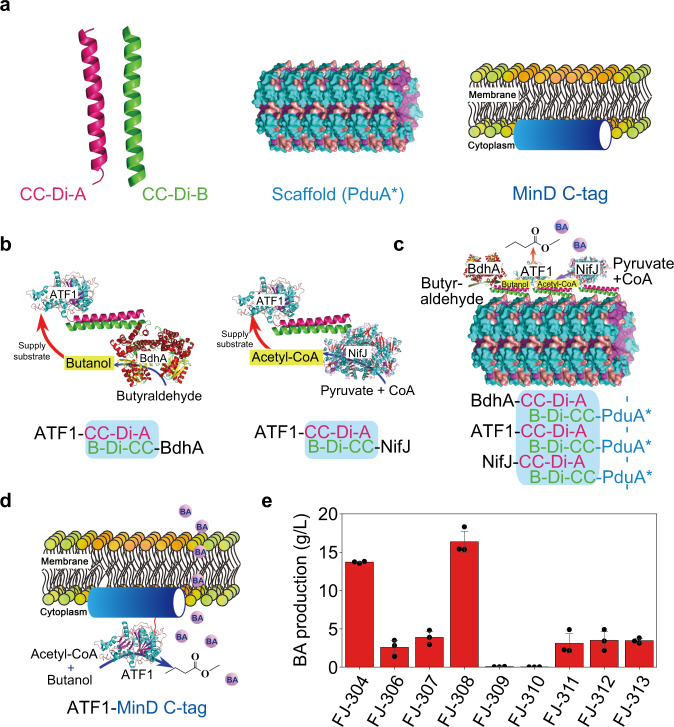
Enhancing butyl acetate production through rational organization of the enzymes associated with BA synthesis. **a** Biological components evaluated in this study for rational organization of enzymes: CC-Di-A and CC-Di-B tags, PduA* scaffold, and MinD C-tag. **b** Schematic representation of assembling two of the three enzymes for BA synthesis with the CC-Di-A and CC-Di-B tags. **c** Schematic representation of organizing the three enzymes for BA synthesis onto the PduA* formed scaffold. **d** Schematic representation of using the MinD C-tag to draw ATF1 onto the cell membrane. **e** The fermentation results for butyl acetate (BA) production with various mutant strains corresponding to the genetic manipulations from (**b**–**d**). Data are presented as mean values ± SD (*n* = 3). Source data underlying Fig. 4e are provided as a Source Data file.

Rational organization of enzymes associated with the synthesis of target product is an effective strategy to improve the bioproduction^38^. In this study, we evaluated several such approaches to increase BA production (Fig. 4).

PduA* protein, derived from *Citrobacter freundii* Pdu bacterial microcompartment could form filaments in bacteria like *E. coli*^38^. The CC-Di-A and CC-Di-B are designed parallel heterodimeric coiled coils and two proteins with each of these self-assembling tags could combine and shorten the catalytic distance. The enzymes (from the same metabolic pathway), tagged with one of the coiled coils (CC-Di-A or CC-Di-B) would attach onto the formed intracellular filaments (its PduA* was tagged by the other coiled coil); thus, the organized enzymes on the filaments would improve the catalytic efficiency of the target metabolic pathway. MinD is a membrane-associated protein and the localization of MinD is mediated by an 8-12 residue C-terminal membrane-targeting sequence. The proteins with MinD C-terminal sequence were able to be drawn to the cell membrane^38,39^. Thus, the application of MinD C-tag can facilitate the secretion of target product and enhance its production by mitigating the intracellular toxicity as well as promoting the catalyzing process (Fig. 4a).

To evaluate whether the organization of enzymes could improve BA production in our strain, three strategies were recruited: (1) assembling the enzymes associated with BA synthesis: NifJ (related to acetyl-CoA synthesis), BdhA (related to butanol synthesis) and ATF1 with the CC-Di-A and/or CC-Di-B tags (FJ-306, FJ-309 and FJ-310); (2) organizing the three enzymes onto PduA* formed scaffold (FJ-311, FJ-312 and FJ-313); or (3) introducing MinD C-tag to draw ATF1 onto the cell membrane (FJ-307, FJ-308).

Firstly, we assembled enzymes for BA synthesis by adding the CC-Di-A tag to ATF1 and the CC-Di-B tag to NifJ and BdhA (Fig. 4b). Fermentation results showed that the addition of CC-Di-A tag to the C-terminus of ATF1 in FJ-306 had significantly negative effects on BA synthesis with only 2.6 g/L BA was produced (Fig. 4e). The assembly of ATF1 together with NifJ, or NifJ and BdhA, had even severer negative effects on BA synthesis and BA production was only 0.06 and 0.03 g/L in FJ-309 and FJ-310, respectively. Further, we organized ATF1, NifJ and BdhA onto the PduA* scaffold. PduA* was tagged with CC-Di-B, while the other three enzymes were tagged with CC-Di-A (Fig. 4c). The generated FJ-311 (harboring the scaffold and ATF1-CC-Di-A) produced 3.1 g/L BA, while FJ-312 (harboring the scaffold, ATF1-CC-Di-A and NifJ-CC-Di-A) and FJ-313 (harboring the scaffold, ATF1-CC-Di-A, NifJ-CC-Di-A and BdhA-CC-Di-A) produced slightly higher amount of BA both at 3.5 g/L. The scaffold seemed to have some positive effects on BA synthesis but didn’t work as efficient as it was reported in other studies^38^. We speculate that the coiled coils tags (CC-Di-A or CC-Di-B) might severely impair the catalytic activity of ATF1. Furthermore, the assembly of ATF1 together with NifJ, or NifJ and BdhA, could further inhibit the activity of ATF1 and significantly impair the cell growth (FJ-309 and FJ-310 demonstrated lowest cell growth among all these strains; see Supplementary Note 3 and Supplementary Fig. 4), thus resulting in significant decrease in BA production in the corresponding strains.

Moreover, we evaluated the effect of the introduction of MinD C-tag (to draw ATF1 onto the cell membrane) on BA production (Fig. 4d). Fermentation results showed that the corresponding FJ-308 strain could produce 16.4 g/L BA, which was 20% higher than FJ-304 (Fig. 4e). The FJ-307 strain (with CC-Di-A-ATF1-MinD) could also produce higher BA of 3.9 g/L compared to FJ-306 (2.6 g/L). All these results indicated that the addition of the cell membrane-associated motif to draw ATF1 onto the cell membrane could facilitate the BA excretion and mitigate the intracellular toxicity and therefore enhance BA production (Supplementary Note 3, Supplementary Fig. 5, and Supplementary Method 3). However, the assembly of BA synthetic enzymes would significantly decrease BA production.

### Elimination of prophages

**Fig. 5:**
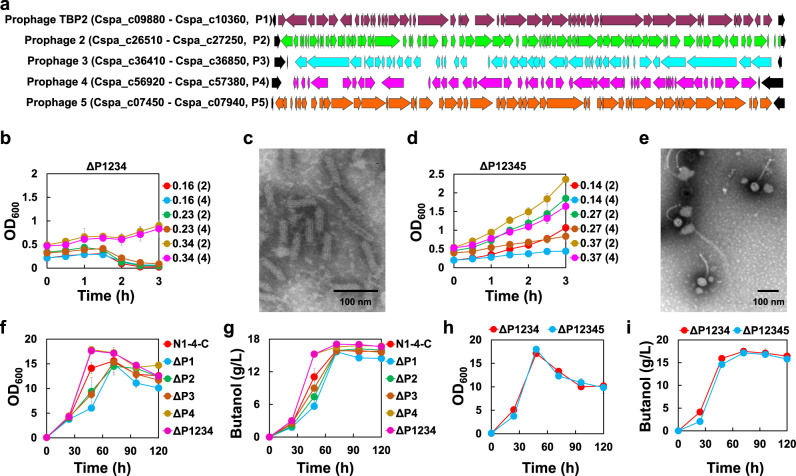
The deletion of the prophage genomes. **a** The gene cluster organization of the TBP2 prophage (P1) and other four putative prophages (P2-P5). **b** Cell growth profiles of ∆P1234 with the induction (at various OD_600_) using mitomycin C at 2 or 4 µg/mL. The value at the right side of the cell growth profile figure represents the actual OD_600_ value at which mitomycin C (with the applied concentration included in the parentheses) was added for the induction. Data are presented as mean values ± SD (*n* = 3). **c** Transmission electron microscopy image of Clostocin O (observed in the supernatant of ∆P1234 after induction with mitomycin C). Similar results were observed in six independent experiments. **d** Cell growth profiles of ∆P12345 with the induction (at various OD_600_) using mitomycin C at 2 or 4 µg/mL. The value at the right side of the cell growth profile figure represents the actual OD_600_ value at which mitomycin C (with the applied concentration included in the parentheses) was added for the induction. Data are presented as mean values ± SD (*n* = 3). **e** Transmission electron microscopy image of the phage TBP2 particles (observed in the supernatant of ∆P2345 after induction with mitomycin C). Similar results were observed in two independent experiments. **f** Comparison of the cell growth of prophage deleted mutants and the control N1-4-C strain. Data are presented as mean values ± SD (*n* = 3). **g** Comparison of the butanol production in prophage deleted mutants and the control N1-4-C strain. Data are presented as mean values ± SD (*n* = 3). **h** Comparison of the cell growth between ∆P1234 and ∆P12345. Data are presented as mean values ± SD (*n* = 3). **i** Comparison of butanol production between ∆P1234 and ∆P12345. Data are presented as mean values ± SD (*n* = 3). Source data underlying Fig. 5b–i are provided as a Source Data file.

During our fermentations, we noticed that the ester production of the strains was not stable and could be varied from batch to batch. It has been reported decades ago that the former strain N1-4 (ATCC 13564) was lysogenized with a temperate phage HM T, generating a new strain N1-4 (HMT), which could release phage particles from the chromosome even without induction^40^. In addition, the N1-4 (HMT) strain can produce a phage-like bacteriocin Clostocin O upon the induction with mitomycin C^41^. We hypothesized that the instability of fermentations with *C. saccharoperbutylacetonicum* might be related to the prophages, and the deletion of prophages would enable more stable and enhanced production of desired endproducts. Our in silico analysis of the N1-4 (HMT) genome revealed five prophage-like regions (referred here as P1-P5, respectively) within the chromosome (Fig. 5a)^42^. Based on systematic evaluation through individual and combinatory deletion of the prophages, we demonstrated that P5 does not have a gene encoding integrase as an intact phage does and is responsible for the Clostocin O synthesis (Fig. 5b & c, and Supplementary Fig. 6; Supplementary Note 4, and Supplementary Methods 4–5)^41^, and further, we confirmed that P1 represents the lysogenic phage TBP2 genome^43^ (Fig. 5d & e, and Supplementary Fig. 7). However, interestingly, the phage image we obtained was different from what was described before. The TBP2 phage we observed in this study consists of a head of roughly 50 nm wide and a tail of about 150 nm long (Fig. 5e & Supplementary Fig. 7); while the phage presented in the image by Schüler et al. comprises a 360 nm long tail as well as a base structure with tail tube and tail fibers^43^. Both ΔP1234 (with the deletion of P1-P4) and ΔP12345 (with the deletion of P1-P5) exhibited improved cell growth, enhanced butanol production, and prolonged cell integrity during fermentation (Fig. 5f–i, Supplementary Note 4, Supplementary Figs. 8, 9, 10 & 11, and Supplementary Method 6). ∆P12345 should be a more stable platform as there is no cell lysis at any induction conditions with mitomycin C (Fig. 5d; Supplementary Note 4, Supplementary Figs. 12 & 13). While ∆P1234 showed similar growth and even slightly higher butanol production compared to ∆P12345 (Fig. 5h & i).

**Fig. 6:**
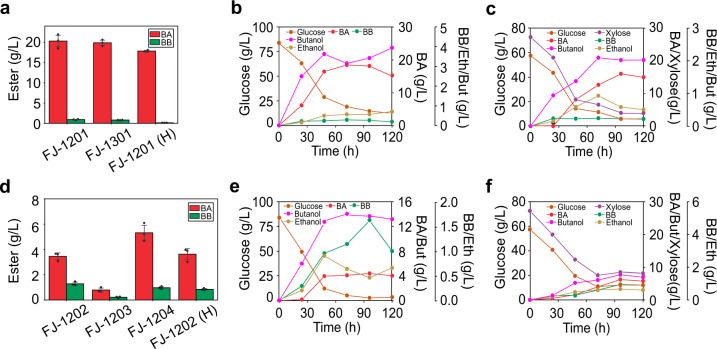
Fermentation results of the engineered strains for fatty acid ester production. **a** BA production in serum bottles using glucose (FJ-1201 and FJ-1301) or biomass hydrolysates (FJ-1201 (H)) as the substrate. Data are presented as mean values ± SD (*n* = 3). **b** BA fermentation kinetics in FJ-1201 in 500 mL bioreactor using glucose as the substrate. **c** BA fermentation kinetics in FJ-1201 in 500 mL bioreactor using biomass hydrolysates as the substrate. **d** BB production in serum bottles using glucose (FJ-1202, FJ-1203, and FJ-1204) or biomass hydrolysates (FJ-1202 (H)) as the substrate. Data are presented as mean values ± SD (*n* = 3). **e** BB fermentation kinetics in FJ-1202 in 500 mL bioreactor using glucose as the substrate. **f** BB fermentation kinetics in FJ-1202 in 500 mL bioreactor using biomass hydrolysates as the substrate. For fermentations with bioreactors, a single set of fermentation was initially carried out with each of the engineered strains due to the limited availability of biomass hydrolysates. When more biomass hydrolysates became available, the composition (primarily the glucose:xylose ratio) of the new biomass hydrolysates was very different from the previous ones that were used; thus, no further replication of the fermentations with bioreactors was conducted. BA: butyl acetate, BB: butyl butyrate, Eth: ethanol, But: butanol. Source data are provided as a Source Data file.

Thus, in a further step, we used ΔP1234 and ΔP12345 as the platform to be engineered for enhanced and more stable BA production. We deleted *Cspa_c47560* and integrated *sadh*-*hydG* cluster in both ΔP1234 and ΔP12345, and obtained FJ-1200 and FJ-1300 correspondingly. The plasmid pMTL-P_*adh*_-*atf1-MinD* was transformed into FJ-1200 and FJ-1300, generating FJ-1201 and FJ-1301, respectively. Fermentation results showed that FJ-1201 and FJ-1301 produced 19.7 g/L and 19.4 g/L BA, respectively, which were both higher than FJ-308 (Fig. 6a & Supplementary Table 1). BA production in both FJ-1201 and FJ-1301 could be completed within 48 h, resulting in a productivity of ~0.41 g/L/h, which was significantly higher than 0.23 g/L/h in FJ-308. BA yield in FJ-1201 reached 0.26 g/g, which was also higher than FJ-308 (0.24 g/g). We further determined BA concentrations in the fermentation broth as 0.6 g/L and 0.5 g/L respectively for the fermentation with FJ-1201 and FJ-1301. Taken together, total BA production in FJ-1201 was 20.3 g/L. It is 2400-fold higher than that has been previously reported^25^, and also 14.8-fold higher than that by the very recently reported *C. diolis* strain^27^.

Besides BA production, BB production in FJ-1201 also reached 0.9 g/L, which was significantly higher than in FJ-308 (0.01 g/L) and in *C. pasteurianum* J-5 (0.3 g/L) (Fig. 2b, Fig. 6a, & Supplementary Table 1). This level (0.9 g/L) was 18.6-fold higher than the BB production that has been previously reported (0.05 g/L in *C. acetobutylicum*)^25^. All these results confirmed our hypothesis that the elimination of prophages would make more robust host strains for enhanced and more stable ester production.

### Expressing SAAT in FJ-1200 further enhanced BB production

As demonstrated in Fig. 2b, SAAT and EHT1 were more relevant for BB production. Therefore, to achieve higher BB production, we expressed *saat* and *eht1* in FJ-1200 and obtained FJ-1202 and FJ-1203, respectively. Fermentation showed that FJ-1202 and FJ-1203 produced 1.3 and 0.2 g/L BB, respectively (Fig. 6d). Further, we added MinD C-tag to the SAAT and introduced the recombinant gene into FJ-1200 and obtained FJ-1204 for an attempt to further improve BB production as observed for BA production in FJ-308. However, BB production in FJ-1204 was only 1.0 g/L. Notwithstanding, 1.3 g/L BB obtained in FJ-1202 is 25.8-fold higher than the level that has been previously reported^25^.

### Ester production with biomass hydrolysates as substrate

Fermentations were carried out using biomass hydrolysates as the substrate. In the hydrolysates, besides sugars (57.4 g/L glucose and 27.2 g/L xylose) as carbon source, there were also nutrients converted from biomass (corn stover). Therefore, we tested the effect of organic nitrogen (yeast extract and tryptone) of various levels on ester production. Interestingly, results showed that the BA production of 17.5 g/L was achieved in FJ-1201 (in the extractant phase) without any exogenous nitrogen source supplemented (Supplementary Table 2). In addition, 0.3 g/L BA was detected in aqueous phase, making a total BA production of 17.8 g/L in FJ-1201 (Fig. 6a & Supplementary Table 2). Although this was slightly lower than when glucose was used as substrate (20.3 g/L), fermentation with hydrolysates did not need any supplementation of nutrients, which could significantly reduce production cost. We further performed fermentation in 500 mL bioreactor with pH controlled >5.0. BA production reached 16.0 g/L with hydrolysates and 18.0 g/L with glucose as substrate, both of which were lower than results from fermentation under the same conditions but with serum bottles (Fig. 6b & c). The lower levels of BA production in larger scale bioreactors (compared to that in serum bottles) could be attributed to various reasons, including but not limited to the architecture of the reactor, the agitation (both mode and speed), and the pH control. More systematic optimization of the fermentation in the bioreactor needs to be carried out to further enhance BA production at larger scales.

Furthermore, we performed fermentation with FJ-1202 for BB production using hydrolysates in both serum bottles and 500 mL bioreactors. Results demonstrated that BB production in serum bottle from hydrolysates was 0.9 g/L (compared to 1.3 g/L when glucose used as substrate; Fig. 6d). BB production in bioreactor from hydrolysates reached 0.9 g/L compared to 1.6 g/L when glucose was used as substrate (Fig. 6e & f). The results were consistent with the case for BA production that lower-level BB was obtained when hydrolysates (compared to glucose) was used as substrate. However, interestingly, larger scale fermentation with bioreactor produced slightly higher level of BB than the fermentation under the same conditions with serum bottle, which was different from the case for BA production.

Carbon balance analyses were performed based on fermentation results for the production of BA in FJ-1201 and production of BB in FJ-1202 (Supplementary Data 1). Carbon recovery rate in percentage was defined as total product carbon divided by total substrate carbon multiplied by 100^44^. Results indicated that when glucose was used as the carbon source, the carbon recovery rate for FJ-1201 is 100.5% in serum bottle fermentation and 101.8% in bioreactor fermentation, respectively; while the carbon recovery rate for FJ-1202 is 89.4% in serum bottle fermentation and 98.3% in bioreactor fermentation, respectively. When biomass hydrolysates were used as the carbon source, the carbon recovery rate for FJ-1201 is 93.1% in serum bottle fermentation and 95.0% in bioreactor fermentation, respectively; while the carbon recovery rate for FJ-1202 is 87.8% in serum bottle fermentation and 95.6% in bioreactor fermentation, respectively.

### Techno-economic analysis for BA production

**Fig. 7:**
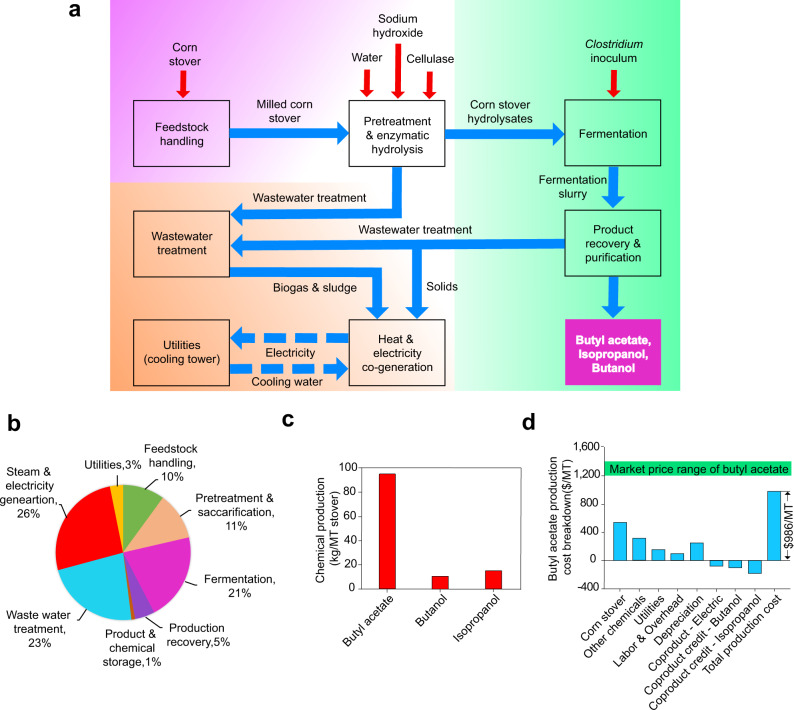
Techno-economic analysis of butyl acetate production from corn stover. **a** Process overview. **b** Total installed equipment cost. **c** Chemical production from each metric tonne (MT) of corn stover. **d** Butyl acetate production cost. Source data underlying Fig. 7b–d are provided as a Source Data file.

We performed a techno-economic analysis (TEA) to evaluate the economic competitiveness of BA production from corn stover at a process capacity of 2500 MT wet corn stover (20% moisture) per day. The whole process was developed based on the previous process using the deacetylation and disk refining (DDR) pretreatment to produce corn stover hydrolysates^45^, which was the substrate used for our fermentation experiments to produce BA. The detailed process information is summarized in Supplementary Method 7. The process is composed of eight sections including feedstock handling, pretreatment and hydrolysis, BA fermentation, product recovery (distillation), wastewater treatment, steam and electricity generation, utilities, and chemical and product storage (Fig. 7a). The detailed material balance, key operation parameters, purchased price of raw materials and utilities and the selling price of co-products (butanol, IPA, and surplus electricity) were shown in Supplementary Tables 3, 4 & 5. The process flow diagram of product recovery for TEA was shown in Supplementary Fig. 14. Figure 7b shows the equipment cost distribution of each process sections, with a total installed equipment cost of $263 million. The fermentation, steam & electricity co-generation, and wastewater treatment contribute significant percentage to the total installed equipment cost, which aligns well with previous TEA models for chemical production from biomass via fermentation^46,47^. It is worth noting that the high installed cost for the wastewater treatment ($59.8 million, Supplementary Table 6) is mainly attributed to the high costs of major equipment, including anaerobic digester, aeration digester, and membrane reactors, used to treat wastewater into clean water that can be recycled back to the system (see details about the process description in Supplementary Method 7). Previous studies on the lignocellulosic biofuel process with a similar capacity showed that the installed cost of wastewater treatment facility was about $50 million^45,46,48^, which is very close to the estimated value in this study. The total capital investment is $472 million by taking consideration of additional direct cost, indirect cost as well as working capitals (Supplementary Table 6). From the process model, 95.2 kg of BA can be produced from 1 MT of corn stover, meanwhile significant amounts of butanol (11.1 kg), isopropanol (15.5 kg) and surplus electricity (209 kWh) are produced as co-products (Fig. 7c). The BA production cost was estimated to be $986/MT (Fig. 7d) with the detailed calculation shown in Supplementary Table 3. This production cost is much lower than the current BA market price ranging between $1200 and $1400 per MT in year 2019 (based on the quotes from the industry^49^), showing the high economic competitiveness of BA production using our engineered strain. By looking into the cost breakdown, the corn stover feedstock cost contributes the most (38.2%) to the BA production cost, followed by other chemicals (22.3%) and capital deprecation (18.0%) and utilities (14.5%). Sensitivity analysis shows that corn stover price, BA yield, and BA titer are the most sensitive input parameters to the BA production cost (Supplementary Fig. 15).

## Discussion

Although tremendous efforts have been invested on biofuel/biochemical research worldwide, very limited success has been achieved. Our central hypothesis is that metabolically engineering of microorganisms for high-value and easy-recoverable bioproduct production (which can lead to high titer and productivity) could be a feasible route to establish highly efficient and economically viable biofuel/biochemical production processes. Here we tested this hypothesis by engineering solventogenic clostridia for efficient ester (high-value and easy-recoverable) production. Our group have previously established versatile genome engineering tools for clostridia^15,18,33^, putting us at a strong position to perform this study.

Based on the systematic screening of host strains and enzymes as well as multiple rounds of rational metabolic engineering: enriching precursors (alcohols and acetyl-CoA) for ester production, dynamically expressing heterologous ester-production pathways, rationally organizing ester-synthesis enzymes, and improving strain robustness by eliminating putative prophages, we ultimately obtained strains for efficient production of esters in both synthetic fermentation medium and biomass hydrolysates (Supplementary Note 5, Supplementary Table 7, and Supplementary Discussion). To the best of our knowledge, the production levels of BA and BB we achieved set up the records. Overall, we demonstrate that clostridia are excellent platforms for valuable biofuel and biochemical production. The general principles that we demonstrated herein, including (1) selecting the most appropriate host for targeted bioproduction and (2) engineering the host for producing high value and easily recoverable products, are highly applicable to other relevant bioprocesses, and may result in breakthroughs in biofuel/biochemical production and general bioeconomy.

## Methods

### Microorganisms and cultivation conditions

All the strains used in this study are listed in Supplementary Data 2. *C. pasteurianum* ATCC 6013 and *C. saccharoperbutylacetonicum* N1-4 (HMT) (DSM 14923) were requested from American Type Culture Collection (ATCC) and Deutsche Sammlung von Mikroorganismen und Zellkulturen (DSMZ), respectively. *C. beijerinckii* NCIMB 8052 was provided by Dr. Hans P. Blaschek^18^. *C. tyrobutyricum* ∆*cat1*::*adhE1* and *C. tyrobutyricum* ∆*cat1*::*adhE2* are hyper-butanol producing mutants constructed in our lab^15^. All the clostridial strains were grown in an anaerobic chamber (N_2_-CO_2_-H_2_ with a volume ratio of 85:10:5) at 35 °C. Strains of *C. tyrobutyricum*, *C. saccharoperbutylacetonicum* and *C. beijerinckii* were cultivated using tryptone-glucose-yeast extract (TGY) medium^50^, while strains of *C. pasteurianum* were cultivated using 2 × YTG medium^51^. When required, clarithromycin (Cla) or thiamphenicol (Tm) was supplemented into the medium at a final concentration of 30 µg/mL and 15 µg/mL, respectively. *E*. *coli* DH5α was used for routine plasmid propagation and maintenance. *E. coli* CA434 was used as the donor strain for plasmid conjugation for *C. tyrobutyricum*. Strains of *E. coli* were grown aerobically at 37 °C in Luria-Bertani (LB) medium supplemented with 100 µg/mL ampicillin (Amp), 50 μg/mL kanamycin (Kan) or 34 µg/mL chloramphenical (Cm) as needed.

### Plasmid construction

All the plasmids used in this study are listed in Supplementary Data 2, and all the primers used in this study are listed in Supplementary Data 3.

The plasmids pMTL82151 and pTJ1 were used for heterogeneous gene expression^50,52^. The promoter of the *cat1* gene (*CTK_C06520*) (P_*cat*_) and the promoter of the *thl* gene (*CTK_C01450*) (P_*thl*_) from *C. tyrobutyricum* ATCC 25755 were amplified and inserted into pMTL82151 at the *Eco*RI site, and the generated plasmids were named as pMTL82151-P_*cat*_ and pMTL82151-P_*thl*_, respectively. Promoters of the following gene, *pflA* (*Cspa_c13710*) (P_*pfl*_), *ald* (*Cspa_c56880*) (P_*ald*_), *adh* (*Cspa_c04380*) (P_*adh*_) and *bdh* (*Cspa_c56790*) (P_*bdh*_), all from *C. saccharoperbutylacetonicum* N1-4 (HMT) were amplified and inserted into pMTL82151 at the *Eco*RI site, and the generated plasmids were named as pMTL82151-P_*pfl*_, pMTL82151-P_*ald*_, pMTL82151-P_*adh*_ and pMTL82151-P_*bdh*_, respectively.

The *vaat* gene from *Fragaria vesca*, the *saat* gene from *F. ananassa* and the *atf1* gene from *S. cerevisiae* were amplified from plasmids pDL006, pDL001 and pDL004, respectively^2,24^. The *atf1’* (the codon optimized *atf1* gene), *eht1* from *S. cerevisiae*^22^, and *lipaseB* from *Candida antarctica*^53^ were all synthesized by GenScript (Piscataway, NJ, USA). The obtained gene fragments of *vaat*, *saat*, *atf1*, *atf1’, eth1*, and *lipaseB* were inserted between the *Btg*ZI and *Eco*RI sites in pMTL82151-P_*cat*_, generating pMTL-P_*cat*_-*vaat*, pMTL-P_*cat*_-*saat*, pMTL-P_*cat*_-*atf1*, pMTL-P_cat_-*atf1*’, pMTL-P_cat_-*eht1*, and pMTL-P_cat_-*lipaseB*, respectively. The *atf1* gene was inserted between the *Btg*ZI and *Eco*RI sites in pMTL82151-P_*thl*_, generating pMTL-P_*thl*_-*atf1*.

The P_*cat*_ promoter and the gene fragments of *vaat*, *saat*, *atf1*, *eth1*, and *lipase* were amplified and ligated into the *Eco*RI site of pTJ1, generating pTJ1-P_*cat*_-*vaat*, pTJ1-P_*cat*_-*saat*, pTJ1-P_*cat*_-*atf1*, pTJ1-P_*cat*_-*eht1* and pTJ1-P_*cat*_-*lipaseB*, respectively. The *atf1* gene was inserted into the *Eco*RI site of pMTL82151-P_*pfl*_, pMTL82151-P_*ald*_, pMTL82151-P_*adh*_ and pMTL82151-P_*bdh*_, generating pMTL-P_*pfl*_-*atf1*, pMTL-P_*ald*_-*atf1*, pMTL-P_*adh*_-*atf1* and pMTL-P_*bdh*-_*atf1*, respectively.

DNA fragments of *CC-Di-A*, *CC-Di-B*, *MinD* and pduA* were synthesized by GenScript (Piscataway, NJ, USA). The MinD-tag was fused to the end of *atf1* with PCR and ligated into the *Eco*RI site of pMTL82151-P_*adh*_, generating pMTL-P_*adh*_-*atf1*-*MinD*. In addition, the MinD-tag was fused to the end of *saat* with PCR and inserted between the *Btg*ZI and *Eco*RI sites of pMTL82151-P_*cat*_, generating pMTL-P_*cat*_-*saat*-*MinD*.

The synthesized *CC-Di-A* fragment was ligated into the *Eco*RI site of pMTL82151-P_*adh*_, generating pMTL-P_*adh*_-CC-Di-A. The DNA fragments of *atf1* and *atf1*-*MinD* were amplified from pMTL-P_*adh*_-*atf1* and pMTL-P_*adh*_-*atf1*-*MinD* and then inserted into the *Eco*RI site of pMTL-P_*adh*_-CC-Di-A, obtaining pMTL-P_*adh*_-A-*atf1* and pMTL-P_*adh*_-A-*atf1*-*MinD*. The *CC-Di-B* sequence with the *nifJ* gene and *CC-Di-B* with the *bdhA* gene were subsequently inserted into the *Kpn*I site of pMTL-P_*adh*_-A-*atf1*, generating pMTL-P_*adh*_-A-*atf1*-B-*nifJ* and pMTL-P_*adh*_-A-*atf1-*B-*nifJ-*B-*bdhA*. The DNA fragments of *CC-Di-B*-pduA*, *CC-Di-A*-*nifJ*, *CC-Di-A*-*bdhA* were inserted into the *Eco*RI site of pTJ1-P_*cat*_, generating pTJ1-P_*cat*_-B-pduA*, pTJ1-P_*cat*_-B-pduA*-A-*nifJ* and pTJ1-P_*cat*_-B-pduA*-A-*nifJ*-A-*bdhA*, respectively.

For the gene deletion or integration in *C. saccharoperbutylacetonicum*, all the relevant plasmids were constructed based on pYW34, which carries the customized CRISPR-Cas9 system for genome editing in *C. saccharoperbutylacetonicum*^18,33^. The promoter P_J23119_ and the gRNA (with 20-nt guide sequence targeting on the specific gene) were amplified by two rounds of PCR with primers N-20nt/YW1342 and YW1339/YW1342 (N represents the targeted gene)^33^. The obtained fragment was then inserted into pYW34 (digested with *Btg*ZI and *Not*I) through Gibson Assembly, generating the intermediate vectors. For gene deletion, the fragment containing the two corresponding homology arms (~500 bp for regular gene deletion; 1000–2000 bp for prophage deletion) for deleting the specific gene through homologous recombination was amplified and inserted into the *Not*I site of the obtained intermediate vector as described above, generating pYW34-∆N (N represents the targeted gene) (Supplementary Fig. 16). For gene integration, the fragment containing the two corresponding homology arms (~1000 bp for each), the promoter and the gene fragment to be integrated, was amplified and inserted into the *Not*I site of the obtained intermediate vector as described above, generating the final plasmid for gene integration purpose. The details about plasmid transformation and mutant verification have been described in Supplementary Method 8.

### Fermentation with glucose or glycerol as the substrate

For the fermentation for ester production, the *C. pasteurianum* strain was cultivated in Biebl medium^54^ with 50 g/L glycerol as the carbon source at 35 °C in the anaerobic chamber. When the OD_600_ reached ~0.8, the seed culture was inoculated at a ratio of 10% into 100 mL of the same medium in a 250 mL serum bottle and then cultivated at an agitation of 150 rpm and 30 °C (on a shaker incubator) for 72 h. The *C. beijerinckii* strain was cultivated in TGY medium until the OD_600_ reached ~0.8. Then the seed culture was inoculated at a ratio of 5% into 100 mL P2 medium along with 60 g/L glucose and 1 g/L yeast extract in a 250 mL serum bottle. The fermentation was carried out at an agitation of 150 rpm and 37 °C for 72 h^50^. The *C. tyrobutyricum* strain was cultivated in RCM medium at 35 °C until the OD_600_ reached ~1.5. Then the seed culture was inoculated at a ratio of 5% into 200 mL fermentation medium (containing: 50 g/L glucose, 5 g/L yeast extract, 5 g/L tryptone, 3 g/L (NH_4_)_2_SO_4_, 1.5 g/L K_2_HPO_4_, 0.6 g/L MgSO_4_ ∙ 7H_2_O, 0.03 g/L FeSO_4_ ∙ 7H_2_O, and 1 g/L L-cysteine) in a 500 mL bioreactor (GS-MFC, Shanghai Gu Xin biological technology Co., Shanghai, China) and the fermentation was carried out at an agitation of 150 rpm and 37 °C for 120 h with pH controlled >6.0^9^. The *C. saccharoperbutylacetonicum* strain was cultivated in TGY medium at 35 °C in the anaerobic chamber until the OD_600_ reached ~0.8. Then the seed culture was inoculated at a ratio of 5% into 100 mL P2 medium along with 80 g/L glucose and 6 g/L tryptone, and 2 g/L yeast extract in a 250 mL serum bottle^17^. The fermentation was carried out at an agitation of 150 rpm and 30 °C for 120 h^50^. For the fermentation at larger scales in bioreactors, it was carried out in a 500 mL fermenter (GS-MFC, Shanghai Gu Xin biological technology Co., Shanghai, China) with a working volume of 250 mL with pH controlled >5.0, at 50 rpm and 30 °C for 120 h. Samples were taken every 24 h for analysis.

For all fermentations in the serum bottle, a needle and hosepipe were connected to the top of bottle for releasing the gases produced during the fermentation. For all the fermentations for ester production, the extractant *n*-hexadecane was added into the fermentation with a ratio of 1:1 (volume of the extractant vs. volume of fermentation broth) for in situ ester extraction. The reported ester concentrations were the determined values in the extractant phase.

### Fermentation with biomass hydrolysates as the substrate

The biomass hydrolysates was kindly provided by Dr. Daniel Schell from National Renewable Energy Laboratory (NREL), which was generated from corn stover through the innovative deacetylation and mechanical refining in a disc refiner (DDR) approach^55^. For the fermentation, the biomass hydrolysate was diluted and supplemented into the P2 medium as the carbon source (with final sugar concentrations of 57.4 g/L glucose and 27.2 g/L xylose). In addition, various concentrations of yeast extract (Y, g/L) and tryptone (T, g/L) were also added as the nitrogen source to evaluate their effects on the fermentation performance: 0Y + 0T; 1Y + 3T and 2Y + 6T. The fermentation was carried out under the same conditions as described above at 100 mL working volume in a 250 mL serum bottle.

### Analytical methods

Concentrations of acetone, ethanol, butanol, isopropanol, acetic acid, butyric acid and glucose were measured using a high-performance liquid chromatography (HPLC, Agilent Technologies 1260 Infinity series, Santa Clara, CA) with a refractive index Detector (RID), equipped with an Aminex HPX-87H column (Bio-Rad Laboratories, Hercules, CA). The column was eluted with 5 mM H_2_SO_4_ at a flow rate of 0.6 mL/min at 25 °C. The concentration of the ester in the *n*-hexadecane phase was quantified using a gas chromatography-mass spectrometry (GC-MS, Agilent Technologies 6890N, Santa Clara, CA) equipped with an HP-5 column (60 m × 0.25 mm, 0.25 mm film thickness). Helium was used as the carrier gas. The initial temperature of the oven was set at 30 °C for 2 min, followed by a ramp of 10 °C/min to reach 300 °C, and a ramp of 2 °C/min to reach the final temperature of 320 °C, which was then held for 2 min. The detector was kept at 225 °C^9^.

All fermentation data were analyzed using Microsoft Excel 2016 (Microsoft Corporation, WA). Origin 2016 (OriginLab Corporation, MA) and Python 3.7 (Python Software Foundation, DE) were used for statistical analysis.

### Reporting summary

Further information on research design is available in the Nature Research Reporting Summary linked to this article.

## Supplementary information

Supplementary Information

Supplementary Dataset 1

Supplementary Dataset 2

Supplementary Dataset 3

Description of Additional Supplementary Files

Reporting Summary

## Data Availability

Data supporting the findings of this work are available within the paper and its Supplementary Information files. A reporting summary for this article is available as a Supplementary Information file. Source data are provided with this paper.

## Source data

Source Data
