# Supplementary material for: Renewable fatty acid ester production in Clostridium
Source: Nat Commun. 2021 Jul 16;12:4368. doi: 10.1038/s41467-021-24038-3 (PMC8285483; doi:10.1038/s41467-021-24038-3)
Supplement: Supplementary file 1 — Supplementary Information [file 41467_2021_24038_MOESM1_ESM.pdf]

Renewable fatty acid ester production in *Clostridium*

Feng *et al*.

Supplementary Method 1. Measurement of intracellular $NADH/NAD^+$ ratio.

To measure the intracellular $NADH/NAD^+$ ratio of the cell culture (FJ-100 vs. N1-4-C), cells were collected at 40 h during fermentation and disrupted by grinding under liquid nitrogen. The intracellular concentrations of NAD^+ and NADH were then measured using the NAD/NADH Quantification Kit (Sigma-Aldrich, St. Louis, MO, USA) following the manufacturer's instruction. All measurements were performed in triplicate.

Supplementary Method 2. Inhibition of isopropanol vs. acetone on cell growth.

To test the relative inhibition of isopropanol vs. acetone on cell growth, the seed culture of either FJ-100 or FJ-300 was prepared by inoculating the glycerol stock of the culture into TGY medium and growing the culture at 35 °C in the anaerobic chamber. When the OD_{600} reached ~0.8, the seed culture was inoculated at a ratio of 10% into 100 mL of the same medium (with the supplementation of either isopropanol or acetone of various concentrations ranging from 0 to 50 g/L) in a 250-mL serum bottle and then cultivated at an agitation of 150 rpm and 30 °C (on a shaker incubator). The OD_{600} was measured every 4 hours throughout the fermentation.

Supplementary Method 3. Quantification of the intracellular fermentation products.

For quantifying the intracellular fermentation products in FJ-304 and FJ-308, samples were taken at 60 h in the fermentation. Cells from 100 mL fermentation broth were collected to the

bottom of two 50 mL centrifuge tubes through centrifugation at 3,230 x g for 20 min at 4 ºC. Then the cells were washed twice using cold PBS buffer (pH 6.0). Afterwards, the cell pellets were resuspended into 500 µL ddH_2O and transferred into a 2 mL centrifuge tube. Subsequently, 600 µL hexadecane was added. Cell disruption was performed using a Bead Ruptor (OMNI International, NW Kennesaw, GA, USA) with the homogenization speed set at 6.5 m/s (each homogenization cycle was for 20s, for totally 6 cycles). The BA (and other products) extracted into hexadecane was measured using a gas chromatography-mass spectrometry (GC-MS, Agilent Technologies 6890N, Santa Clara, CA) equipped with an HP-5 column (60m×0.25 mm, 0.25 mm film thickness). The total protein concentration in each sample was estimated using a dye-binding method[1], using Bovine serum albumin (BSA) as the standard. The reported intracellular BA concentration was normalized against the total amount of protein in the sample. All measurements were performed in triplicate.

Supplementary Method 4. Prophage induction and phage harvesting.

The strain was grown in TGY medium for overnight. The cells were then transferred into fresh TGY prior to be expose to the inducing reagent mitomycin C or norfloxacin. Mitomycin C was added into the culture when the cell growth reached the desired OD_{600} (0.1-0.2, 0.2-0.3, 0.3-0.4 or 0.4-0.5). The cells of some of the cultures grew too fast at the early stage for the induction purpose, and thus they were not induced under every above mentioned OD_{600} conditions in this work. For the induction, generally mitomycin C at final concentrations of 2 µg/mL and 4 µg/mL was used[2]. Besides, 1 µg/mL and 3 µg/mL of mitomycin C were also tried for the induction in ΔP234. After 30 min of treatment at 35 °C, the cells were harvested via centrifugation at 4,000 g for 5 min and resuspended at the same volume of TGY fresh medium. Then the OD_{600} was monitored carefully during the following 3-5 h.

The cells were collected via centrifugation at 4,000 g for 20 min, and then filtered through the 0.2 μm filter to obtain the supernatants. The supernatants were collected by ultracentrifuge (Sorvall LYNX 6000, ThermoFisher Scientific) at 70,000 g for 3h, and the obtained precipitation (containing the phages) was then resuspended by 1/30 volume of ddH_2O.

Supplementary Method 5. Transmission electron microscopy.

A total of 20 μL prepared phage sample was applied to a mesh copper grid and settled for 2 min. Then the liquid was blotted off with a filter paper. The negative stain of 2% phosphotungstic acid (PTA) was applied to a mesh copper grid and settled for 30 s. Then the liquid was blotted off with a filter paper followed by air dry. The prepared samples were then used for transmission electron microscopy (TEM) observation under a Zeiss EM10 transmission electron microscope (Carl Zeiss AG, Oberkochen, Germany) at an accelerating voltage of 60 kV. The images were acquired and analyzed with Maxlm DL5 software (Cyanogen Imaging, Ottawa, ON, Canada).

Supplementary Method 6. Phase contrast microscopy.

Cell cultures (N1-4-C, ΔP1234, and ΔP12345) were grown in P2 medium using 60 g/L sucrose as the substrate. At specific time points, cell culture samples were harvested by centrifugation, washed twice and resuspended in distilled water. Cell morphology was examined using an Olympus BX53F Upright Microscope (phase contrast mode) equipped with an Olympus DP73 Camera (Olympus Corporation, Shinjuku-ku, Japan).

Supplementary Method 7. Techno-economic analysis.

Modeling overview

A comprehensive TEA model was developed to evaluate the economic feasibility of BA production from sugar hydrolysates from corn stover using the deacetylation and disk refining (DDR) process. The model originally developed to produce ethanol[3] was modified to produce BA by mainly substituting the fermentation and distillation unit operations. The processing capacity was set at 2,500 wet metric tonnes (MT, 20% moisture) of corn stover per day. The process was assumed to run 350 days (8,410 hours) per year; thus, the annual corn stover consumption is 875,000 wet MT per year. The whole process was grouped into eight major sections, namely feedstock handling, DDR pretreatment and hydrolysis, fermentation, product recovery (distillation), steam and electricity cogeneration, wastewater treatment, utilities, and chemical and product storage (Fig. 7a). All process was simulated using the software SuperPro Designer (v11.0, Intelligen Inc., NJ). After completion of the process simulation, the results of mass balance (i.e., materials flow in and out of the different unit operations) and energy balance (i.e., energy in and out of different unit operations) were used to determine the raw materials consumptions, product generations, equipment size and quantity, utility requirements, and other direct and indirect costs associated with capital and operating costs. After identifying the total capital investment, operating costs, and product generation rates, the economic analysis was performed in Microsoft Excel Spreadsheet (v16.38, Microsoft Corporation, WA) to determine the unit production cost of BA.

Descriptions of major processing sections

Feedstock receiving and handling. Corn stover is stored in a central depot, milled to 0.16-0.23 inch, and delivered to the biorefinery plant with uniform-format specifications such as

particle size, moisture content, and bulk density. The composition of corn stover is 35.05% cellulose, 19.53% hemicellulose, 15.76% lignin, 4.93% ash, 3.10% protein, and 21.63% other solids on a dry basis[4]. Trucks for delivering corn stover are weighted by an electronic scale and unloaded using a whole-truck dumper to concrete storage domes, which are sufficient to store a 3-day supply of feedstocks for a weekend buffer. Conveyors are used to transport corn stover to receivers (bins) on the pretreatment reactor. Although feedstock washing has the benefits of removing contaminants (e.g., soils), corn stover is not washed due to the enormous water consumption[4, 5].

Pretreatment and hydrolysis. Pretreatment of corn stover is necessary to reduce the recalcitrant structure of lignocellulosic materials for better enzymatic hydrolysis. The DDR method is applied to the process because the hydrolysates used in the experiments were produced from the corn stover pretreated by the DDR method. Received corn stover is added with water to obtain a 25 wt% solid slurry. The slurry is added with sodium hydroxide at a loading of 40 kg/MT dry corn stover, heated to 80 °C, and held for 2 hours to remove acetyl groups from corn stover. The alkali-treated corn stover is then washed by using the same amount of added water, followed by dewatering using screw presses to remove excess water to attain 40% solids content for the subsequent disk refining. During the disk refining, a Sprout Model 401 91-cm commercial-scale refiner is used to mill corn stover[3]. The disk refiner has two counter rotating disks with a rotating speed at 1,200 rpm. The electricity consumption of disk milling is 212 kWh per dry MT of corn stover. The pressed liquor (from the screw presses) containing spent sodium hydroxide, acetate, and lignin is acidified by sulfuric acid to precipitate lignin used for boil fuels. After lignin removal, the remained liquid is sent to the wastewater treatment section for further processing. The details of the DDR process are described in Chen *et al.*[3].

The pretreated corn stover is mixed with water to make a 20 wt% solid slurry and then cooled to 48 °C before entering hydrolysis tanks. Enzymes (cellulase) are pumped to the hydrolysis tanks at an enzyme loading of 19 mg protein/g cellulose, which is based on the optimization experiments conducted by Chen *et al.*[3]. The added enzymes are well mixed with the corn stover slurry by mechanical agitators and the temperature is maintained with cooling water. The enzymatic hydrolysis takes 84 hours, during which the enzyme breakdown cellulose and hemicellulose into glucose, xylose, and other minor sugars. The hydrolysis yield of cellulose to glucose is assumed to be 84% and the hydrolysis yield of hemicellulose to xylose (and other minor sugars) is assumed to be 74% based on the experimental results from Chen *et al.*[3].

Fermentation Section. After enzymatic hydrolysis, the saccharified slurry is cooled to heat exchangers to 30 °C for fermentation. The engineered *Clostridium* bacterium is used as the culture. 10% of the hydrolysate is split off to seed fermenters for production of seed culture, and the rest 90% is sent to fermenters and converted into BA, butanol, and isopropanol (IPA), where BA is the main product with butanol and IPA as co-products. The seed culture preparation consists of five-stage propagations, with the volume of each stage increasing by 10 times with the details provided by Humbird *et al.*[4]. The first stage reactor (15 gallons (56.8 liters) in size) is inoculated with a seed culture from the lab. After 24 hours of incubation, its broth is used to inoculate the second-stage reactor (150 gallons (568 liters) in size), whose broth is used to inoculate the third-stage reactor, and so on. The broth from the last-stage reactor (~150,000 gallons (567,812 liters) in size) is used to inoculate the large fermenters. At the beginning of the fermentation, hexadecane is added to the fermenters at a 1:1 ratio (v/v) to extract BA from the fermentation broth. The key parameters for the fermentation conditions

and yields are summarized in Supplementary Table 4. The fermentation takes 96 hours at 30 °C to convert all hydrolyzed sugars (72.9 g/L) to BA and coproducts butanol and IPA. Based on our experimental results, the BA, butanol, and IPA yields are set at 0.25, 0.03 and 0.04 g/g consumed sugar, respectively. No external nutrients are added to the fermenters as our experiments showed that nitrogen supplementary does not improve the BA fermentation.

Product Recovery Section. A complex sequence of distillation steps is designed to recover BA, butanol, and IPA and recycle hexadecane from the fermented broth[4, 6, 7]. As shown in Supplementary Fig. 14, fermentation broth (containing BA, butanol, IPA, water, hexadecane, and solids) from the broth surge tank is sent to a decanter unit, where 96% of BA is dissolved in hexadecane floating on the top and the rest 4% of BA is lost in aqueous phase based on our experimental results. Butanol and IPA stay in the aqueous phase due to the high solubility of butanol and IPA in water. Distillation of BA from hexadecane is accomplished in two columns where BA is the light phase and the hexadecane is the heavy phase. The first column distillate BA and removes most of the hexadecane. The second column concentrated the BA to 99.6%. The distillation process is simulated using the non-random two liquids (NRTL) model embedded in the SuperPro software. There is 0.9% of BA lost to the heavy phase (the hexadecane stream); however, since the hexadecane is recycled back to the fermenters, there is minimal loss of the BA during distillation. It is important to note that the repeated use of hexadecane in fermentation has not been verified by the experiments, but it is necessary to verify it in the future. The distillation for the separation of IPA and butanol from the aqueous phase is designed[6-8]. Briefly, the whole aqueous phase from the decanter is sent to the beer column to remove about 90% of the water and all solids from butanol and IPA. The light phase from the beer column contains concentrated butanol and IPA (in water) and is sent to the IPA column. The IPA column distillate IPA, while the heavy phase (butanol-water mixture) is sent

to the 1st butanol column for butanol recovery. Since IPA forms an azeotrope with water at 87.8 wt% and 80 °C (distillation temperature) at atmospheric pressure[9], it is impossible to produce a high purity IPA through the conventional distillation system. Therefore, the IPA at 87.8 wt% purity from the distillation column is further dehydrated to 99.9% by vapor-phase molecular sieve adsorption. During regeneration of the molecular sieve, a low-purity IPA (40%) stream is generated and is sent back to the IPA column for recovery. The butanol separation is designed using a two-column/decanter system suggested by Luyben[6] and Doherty and Malone[10] based on the rigorous modeling on the butanol/water binary system. Both of the columns are stripping columns with their individual reboiler. The first butanol column removes the majority of water and concentrates butanol. The bottom of the column (heavy phase) contains 99.9 wt% water, which is sent to the wastewater section for treatment. The top of the column (light phase) contains 25.8 wt% butanol, which is sent to the decanter. The second butanol column produces 99.8 wt% butanol at the bottom (heavy phase) as the final butanol product. The light phase from both columns is sent to the decanter, in which the aqueous and organic liquid phase separate. The organic phase is sent to the second butanol column whereas the aqueous phase is sent back to the first butanol column. The details of the column design and simulation can be found in Luyben[6]. It is important to note that the whole distillation design only represents one effective design for the recovery of BA, IPA, and butanol, but not necessarily means the most optimal design for the lowest energy consumption. In the future, the further optimization of the distillation system is needed.

Wastewater treatment section. Biomass pretreatment, fermentation, and product recovery sections produce several wastewater streams that must be treated before cycle to the process or release to the environment. The wastewater mainly contains unutilized sugars, processing byproducts (e.g., acetic acids), chemicals, and bacterial cells. The wastewater treatment system

considered in this study was designed according to a process model in a previous study[4], which includes anaerobic digestion, aerobic digestion, sludge dewatering, and membrane bioreactors. Specifically, the wastewater was first sent to an anaerobic digester, where most of the unutilized sugars and short-chain fatty acids (acetic acids) are converted into biogas. The biogas is delivered to the combustor to produce heat and electricity in the cogeneration section. The liquid from the anaerobic digester is delivered to aerobic digesters, where remaining soluble organic matter is converted into carbon dioxide, water, and cell biomass. After the anaerobic and aerobic digestions, it is assumed that more than 99% of soluble organic matter is removed[4, 5]. The treated slurry is then pumped into a clarifier to generate two streams: clear liquid and sludge. The clear liquid is pumped into the membrane bioreactors, which use a series of ultrafiltration and reverse osmosis membranes to further purify water. It was assumed that > 99 wt% pure water is recovered after the membrane system, and the purified water will be recycled to the pretreatment, hydrolysis, and cogeneration sections. The sludges from both anaerobic and aerobic digestions are combined and dewatered using a centrifuge. The centrifuge solids (mainly cell biomass and residual fibers) are burned in the combustor and the centrifuge water is recycled to the aerobic digester for further treatment[4].

Steam and electricity cogeneration. The steam and electricity cogeneration units comprise a combustor and boiler and a turbogenerator to burn various organic streams to produce steam and electricity. The various organic streams include the precipitated lignin during pretreatment, uncovered cellulose and hemicellulose after fermentation and distillation, biogas from anaerobic digestion and dewatered sludge. Such a cogeneration system for burning wet organic stream was designed by the National Renewable Energy Laboratory (NREL)[4] and the process design and technical data from the NREL study were adopted for the simulating of the steam and electricity generation. The wet organic solids with 46% moisture content, together with

biogas, is fed to the combustor to burn to generate heat. The amount of generated heat is calculated by the embedded burner-module in SuperPro Design based on the elemental composition of the organic solids and biogas, assuming 20% of heat loss[11]. The generated heat is used to boil feed-in water into superheated and high-pressure steam at 454 °C and 900 psig[4]. The superheated steam enters the turbogenerator to produce electricity and meanwhile generate steam (350 kPa, 139 °C). The steam is used as the heating agent in pretreatment, distillation, and other processes.

Product and feed chemical storage. This section includes storage bins and containers used for storage products (BA, butanol, and IPA) and feed-in chemicals (e.g., hexadecane, sulfuric acid, alkali, enzymes, and freshwater). The volumes of the storage bins were designed to have a 7 –14 days buffer time.

Process economics

The cost year of 2019 was chosen for this analysis. The energy and mass balance and flow rate information were generated as a basis to determine the size and quantify of equipment for each unit operation. Based on the determined information, the purchased equipment costs were determined based on previous literature, particularly from Humbird *et al.*[4] and Chen *et al.*[3]. The cost of the product recovery (distillation) units and small equipment such as pumps were mainly determined by the embedded cost estimator of SuperPro Designer. In the cases that the equipment size is different from that from literature, an exponential scaling expression was used to estimate the purchase cost of new size equipment:

$$New\ cost = (Base\ Cost) \times (\frac{New\ Size}{Base\ Size})^n \qquad (1)$$

where *n* is a characteristic scaling exponent (0.5-0.7) based on different types of equipment[4]. The equipment cost obtained in previous years is adjusted to the year of 2019 using the plant

cost index from chemical engineering magazine[12]. The equation used for the adjustment is:

$$2019\ Cost = (Base\ Cost) \times \left(\frac{2019\ Cost\ Index}{Base\ Year\ Index}\right) \qquad (2)$$

Besides the equipment purchase cost, the equipment installation cost was added using an installation factor for different types of equipment to obtain the installed equipment cost[13]. The total capital investment (TCI) was calculated as a sum of direct and indirect costs, which were determined based on the installed equipment costs. Direct costs included installed equipment cost, site development (9% of inside-battery-limits (ISBL) equipment cost), warehouse (4.0% of ISBL), and additional piping (4.5% of ISBL). Indirect cost is the sum of prorateable costs (10% of total direct cost (TDC)), field expenses (10% of TDC), home office and construction (20% of TDC), project contingency (10% of TDC), and other costs (10% of TDC). Working capital was assumed to be 5% of the fixed capital investment[4].

The total operating cost included both variable operating costs, including raw material costs, utilities, and co-product credits, and fixed operating costs including labor and various overhead costs. For the variable operating costs, the purchased price of raw materials and utilities and the selling price of co-products (butanol, IPA, and surplus electricity) are listed in Supplementary Table 5. For the fixed operating costs, it is assumed the 50 employees are recruited for operating the plant with an average annual salary of $50,000 per year, which is in alignment with the NREL study. A 90% labor burden is applied to the total salary. The labor burden covers general plant maintenance, payroll overhead, plant security and safety, general engineering, and other services (e.g., phone, light) to keep the plant operating smoothly. Moreover, plant maintenance cost is assumed to be 3% of ISBL and the property issuance is assumed to be 0.7% of fixed operating costs[4]. For calculating the plant depreciation cost, a straight-line depreciation method is used by assuming a 20-year project. Once the total capital investment, variable operating costs, and fixed operating costs have been determined, the BA

production cost was calculated based on the methods described in prior studies[14, 15]. It was assumed that plant would be a 100% equity purchase, so there is no interest charge. Sensitivity analyses were also performed at ± 20% variation range to evaluate the most influential variables on the BA production cost (Supplementary Fig. 15).

Supplementary Method 8. Plasmid transformation and mutant verification.

Competent cells of *C. pasteurianum* were prepared following the protocol as reported by Pyne *et al.*[16]. *C. pasteurianum* SD-1, an equivalent to *C. pasteurianum* Δ*cpaAIR* in which the *cpaAIR* gene (encoding the CpaAI Type II restriction endonuclease) was deleted and thus more efficient transformation was enabled[17], was used as the host strain. The overnight-grown seed culture was inoculated into 20 mL 2×YTG medium. When the OD_{600} reached 0.3~0.4, sucrose and glycine were added to a final concentration of 0.4 M and 1.25%, respectively. When the OD_{600} further reached 0.6~0.8, the cells were harvested by centrifugation at 4,200 g and 4 °C for 10 min. The cell pellets were resuspended in 5 mL of SMP buffer (270 mM sucrose, 1mM $MgCl_2$ and 7mM sodium phosphate, pH 6.5) and spinned down under the same conditions. The obtained cell pellets were then resuspended into 0.6 mL of SMP buffer. 1 μg of plasmid DNA was suspended with 20 μL of 2 mM Tris-HCl (pH 8.0) and then mixed with 550 μL competent cells and 30 μL 96% cold ethanol. The mixture was transferred to a pre-chilled 4 mm electroporation cuvette and incubated on ice for 5 min. Electroporation was then applied with a voltage of 1,800 V, capacitance of 25 μF and resistance of infinity using a Gene Pulser Xcell electroporation system (Bio-Rad Laboratories, Hercules, CA). Afterwards, the culture was transferred into 2 mL of 2×YTG medium and recovered at 35 °C for 4 h. The culture was then spread onto the 2×YTGT agar plates (2×YTG agar plates containing 15 μg/mL of thiamphenicol) for the selection of the transformants.

Competent cells of *C. beijerinckii* were prepared following the procedure as described by Wang *et al.*[18]. Briefly, the overnight cell culture was inoculated into TGY medium with an inoculation ratio of 1%. When the OD_{600} reached ~0.8, the cells were harvested by centrifugation at 4,200 g and 4 °C for 10 min. The cell pellets were washed with the same volume (as the original cell culture) of ice-cold 15% glycerol and centrifugated under the same condition for 10 min. The cell pellets were resuspended with 5% volume of ice-cold 15% glycerol. Then 400 µL competent cells and ~1.0 µg of plasmid DNA were mixed and transferred into a 2-mm pre-chilled electroporation cuvette and incubated on ice for 10 min. Electroporation was carried out at 2,000 V of voltage, 25 µF of capacitance and 200 Ω of resistance. Afterwards, the cells were transferred into 1.6 mL of TGY and incubated at 35 °C for 6-8 h for recovery. The culture was then spread onto TGYC agar plates (TGY agar plates containing 30 µg/mL of clarithromycin) for the selection of transformants.

The plasmid transformation through conjugation for *C. tyrobutyricum* was performed following the procedure as described by Zhang *et al.*[19]. The donor strain *E. coli* CA434 carrying the desired plasmid was cultivated in LB medium supplemented with Cm and Kan. 3 mL of overnight-cultured *E. coli* CA434 cells were centrifuged and washed for twice (with fresh LB medium) to remove the antibiotics. The obtained donor cells were then mixed with 0.4 mL of the overnight-cultured *C. tyrobutyricum* (grown in TGY medium). The cell mixture was spotted onto the TGY agar plate and incubated in the anaerobic chamber at 37 °C for conjugation. After 24 h of cultivation, the cell lawn on the plate was washed off using 1 mL of TGY medium and then spread onto the TGY plate containing 15 µg/mL Tm and 250 µg/mL D-cycloserine (for eliminating the residual *E. coli* CA434 donor cells). The transformant colonies could be observed after 48-72 h of incubation in the anaerobic chamber.

Competent cells of *C. saccharoperbutylacetonicum* were prepared following the procedure as described by Herman *et al.* with slight modifications[20]. Briefly, the overnight-cultured cells were inoculated into fresh TGY medium. When the OD_{600} of the cell culture reached ~0.8, cells were collected by centrifugation at 4,200 g and 22 °C (room temperature) for 10 min. Cell pellets were then washed with the same volume (as the original cell culture) of SMP buffer. The resuspension was centrifuged again under the same conditions as described above. The cell pellets were resuspended in 5% volume of SMP buffer. After that, the plasmid (~1.0 μg) was mixed with 400 μL of competent cells and transferred into a 2 mm electroporation cuvette and incubated on ice for 30 min. Electroporation was then applied with a voltage of 1,000 V, capacitance of 25 μF and resistance of 300 Ω. Subsequently, the culture was transferred into 2 mL pre-warmed TGY medium and incubated at 35 °C for 2-3 h. After that, the culture was spread onto TGYC or TGYT (TGY agar plates containing 15 μg/mL of thiamphenicol) agar plates.

The positive mutants of *C. saccharoperbutylacetonicum* with desired gene deletion or integration were identified following our previously described procedures[21]. Briefly, the *C. saccharoperbutylacetonicum* transformants harboring the plasmid designed for the gene deletion or integration were incubated in TGYC liquid medium at 35 °C in the anaerobic chamber for about 24 h. The cell culture was then spread onto TGYLC plates (TGYC supplemented with 40 mM lactose). When colonies appeared on the plates, colony PCR (cPCR) was then carried out with the pair of primers of N-U/N-D (N represents the targeted gene name, U represents the upstream primer flanking the target locus, and D represents the downstream primer flanking the target locus) to verify the gene deletion or gene integration. The selected mutants were then subcultured in TGY medium for 3 to 5 generations to cure the plasmid for

the gene deletion/integration[21]. The obtained plasmid-free and marker-free mutant strains were used for the following steps.

Supplementary Note 1. Enhancement of NADH availability.

Based on the gene arrangement and the protein sequences of *Cspa_c47560*/*Cspa_c47550*, we deduced that the two genes may encode a heterodimeric oxidoreductase complex, and their functions have no close relation with their neighboring genes based on the annotation. The analysis on the conserved domain showed that the *Cspa_c47550* encoded protein is composed of an NuoF domain (21-349aa) and a DMSOR_beta-like superfamily domain (360-414aa). The NuoF domain can bind an NAD^+, a flavin, and a [4Fe4S]-cluster, and the DMSOR-beta domain can bind two [4Fe4S]-clusters. The *Cspa_c47560* encoded protein is composed of an NuoG domain (2-207aa) and a GltD domain (NADPH-glutamate synthase small subunit, 222-616aa). The NuoG domain can bind a [2Fe2S]-cluster and 3 [4Fe4S]-clusters, and the GltD domain can bind an NADP and a flavin. These domains often exist and play important roles in the recently discovered flavin-based electron-bifurcating NADH-dependent reduced ferredoxin:$NADP^+$ oxidoreductase NfnAB[22, 23], [Fe-Fe]-hydrogenase HydABC[24], formate dehydrogenase HylCBA/FdhF2[25], and [Fe-Fe]-hydrogenase/formate dehydrogenase complex FdhA/HytA-E[26]. However, there is no core domain/subunit identified in the *Cspa_c47560*/*Cspa_c47550* encoded protein complex, for catalyzing reduction or dehydrogenation of a metabolite like [H]-cluster containing HydA and molybdopterin-containing Fdh. Since it can bind NAD^+, $NADP^+$, and most likely also ferredoxin, the enzymatic complex encoded by *Cspa_c47560*/*Cspa_c47550* looks similar to NfnAB in function, catalyzing the transhydrogenation reaction among NAD^+, $NADP^+$, and ferredoxin, although the identity in their protein sequences is actually very low. Indeed, by deleting *Cspa_c47560*, the BA production was significantly improved in FJ-101, confirming that *Cspa_c47560*/*Cspa_c47550* encoded enzymatic complex may function in the direction of NADH dehydrogenation and ferredoxin oxidation; with the deletion of *Cspa_c47560*, NADH and/or reduced ferredoxin are saved and thus utilized for enhanced BA production.

In order to confirm that the deletion of *Cspa_c47560* enhanced NADH availability (or $NADH/NAD^+$ ratio) in the mutant strain and thus improved BA production, we measured the $NADH/NAD^+$ ratio in FJ-100 (in which *Cspa_c47560* was deleted) compared to the mother strain N1-4-C. As shown in Supplementary Fig. 2, the $NADH/NAD^+$ ratio in FJ-100 is indeed significantly higher than that in N1-4-C.

Supplementary Note 2. Tuning of acetyl-CoA availability/flux.

It is surprising that BA production was significantly enhanced when the pathway of converting acetone into isopropanol was introduced. Because based on the pathway, when one additional mole of acetone is produced (by pulling the flux to isopropanol), one more mole of acetoacetyl-CoA will be consumed, which should be generated from two moles of acetyl-CoA, while only one mole of acetyl-CoA will be re-generated accompanying the production of one mole of acetone (and thus the total acetyl-CoA availability would be decreased in this case). However, on the contrary, one seemingly plausible speculation is that, the introduction of the isopropanol pathway to pull the flux from acetoacetyl-CoA for acetone production would not necessarily (or at least not exclusively) further pull the flux from acetyl-CoA for acetoacetyl-CoA production, but rather would diminish the flux of acetoacetyl-CoA towards butanol (and butyrate) production. This is consistent with the fact that the butanol production in FJ-301 decreased to 7.1 g/L (compared to 7.6 g/L in FJ-101) in this study. In this case, the overall acetyl-CoA availability (or flux) could be increased by the enhanced regeneration process due to the conversion of acetone to isopropanol, and thus lead to increased BA production as we observed in FJ-201 and FJ-301. Anyway, to elucidate the exact mechanism for enhanced BA production when the isopropanol-producing pathway was introduced, a systematic metabolic

flux analysis and/or metabolic modelling analysis would be warranted in the following study.

Besides, another possibility could be that isopropanol might be less toxic than acetone to the strain; by converting acetone into isopropanol in FJ-201 and FJ-301, the endproduct toxicity was mitigated, and thus the cell viability (and also BA production) was enhanced. To test this hypothesis, we carried out experiments to assess the relative inhibition of isopropanol vs. acetone (at various concentrations ranging from 0 to 50 g/L) on the cell growth using FJ-100 (without the isopropanol production pathway) and FJ-300 (with the integrated *sadh-hydG* gene cluster for isopropanol production). As shown in Supplementary Fig. 3, in most cases, the cell growth kinetics (for the same strain with the supplementation of the same level of either isopropanol or acetone in the medium) were generally very similar to each other, except that FJ-100 grew to slightly lower OD_{600} when 9, 15, or 50 g/L isopropanol (vs. the corresponding same level of acetone) was supplemented (Supplementary Fig. 3a). Therefore, the above hypothesis was tested as negative.

In the pathway, thiolase is the enzyme that coverts acetyl-CoA into acetoacetyl-CoA (Fig. 3a), and the attenuation of the thiolase activity might be able to repress the acetyl-CoA flux towards butanol (and other products) and lead to the accumulation of intracellular acetyl-CoA, which could be beneficial for enhanced BA production. Therefore, we attempted to delete the thiolase gene to increase the acetyl-CoA availability (Strategy 1), along with Strategy 2 (integrating the pathway of converting acetone into isopropanol; Fig. 3a). There are five annotated genes encoding thiolase in *C. saccharoperbutylacetonicum*: *Cspa_c06180*, *Cspa_c17310*, *Cspa_c20520*, *Cspa_c20890*, *Cspa_c50320*. However, we initially failed to delete any of them despite multiple trials. After FJ-300 was obtained, we further sought to delete these genes. After numerous attempts, we were only able to delete *Cspa_c20890* and

Cspa_c50320 individually in FJ-300, generating the mutant strains FJ-400 and FJ-500, respectively. By introducing the vector pMTL-cat-*atf1* into these two strains for BA production, FJ-401 and FJ-501 were obtained, respectively. Fermentation results indicated that BA production was actually slightly lower in FJ-401 (10.8 g/L) and FJ-501 (9.8 g/L) than that in FJ-301 (Fig. 3b). The result was unexpected. Acetoacetyl-CoA is one of the key metabolites for the cell; the repression for its biosynthesis might impair the cell metabolism and thus lead to decreased BA production.

Supplementary Note 3. Rational organization of BA-synthesis enzymes.

The cell growth profiles of the various mutant strains (with the rational organization of enzymes associated with BA synthesis) were shown in Supplementary Fig. 4. Compared to FJ-304, FJ-308 (with the MinD C-tag to draw ATF1 onto the cell membrane) demonstrated very similar cell growth dynamics, indicating that the expression of MinD C-tag did not significantly influence the cell growth (while significantly improve BA production). While when the CC-Di-A tag was added to ATF1, the cell culture of FJ-306 and FJ-307 (an additional MinD C-tag was included besides the CC-Di-A tag) actually grew to much higher OD (105.1% higher maximum OD in FJ-306 and 83.5% higher maximum OD in FJ-307) than FJ-304. This might be because the addition of CC-Di-A tag to ATF1 led to decreased BA production (Fig. 4e; but did not influence the cell viability by itself) and thus mitigated toxicity and improved cell growth. However, when ATF1 was assembled together with NifJ (in FJ-309), or NifJ and BdhA (in FJ-310), which are genes related to butanol and BA production within the native pathway of the strain, using CC-Di-A and CC-Di-B tags, the cell growth was severely inhibited (53.2% lower maximum OD in FJ-309 and 30.4% lower maximum OD in FJ-310 comparing to that in FJ-304) in correspondence to negligible BA production (Fig. 4e). Further, when ATF1, NifJ

and BdhA was organized onto the PduA* scaffold (PduA* was tagged with CC-Di-B, while the other three enzymes were tagged with CC-Di-A; Fig. 4c), the corresponding strains FJ-311 (ATF1 was tagged with CC-Di-A), FJ-312 (both ATF1 and NifJ were tagged with CC-Di-A), and FJ-313 (ATF1, NifJ, and BdhA were all tagged with CC-Di-A) all demonstrated comparable cell growth as FJ-304, which were much higher than FJ-309 and FJ-310. This suggested that the scaffold might have some positive effects on mitigating BA toxicity in consistence with slightly increased BA production and cell growth (compared to the case in FJ-309 and FJ-310).

We measured the concentrations of the intracellular and extracellular products in FJ-304 and in FJ-308, respectively (at 60 h of the fermentation). As shown in Supplementary Fig. 5, while FJ-308 produced significantly higher extracellular BA than FJ-304, the concentration of intracellular BA in FJ-308 was much lower than that in FJ-304 (the intercellular level of other products including acetate, butyrate, ethanol or butanol was not detectable in either FJ-304 or FJ-308). This very well supports our hypothesis that the application of MinD C-tag to position ATF1 to the cell membrane (in FJ-308) can facilitate the secretion of BA outside of the cell and enhance BA production.

Supplementary Note 4. Elimination of prophages.

During our fermentation process, we noticed that the performance for ester production of the strains was not very stable and could be varied from batch to batch. Similar cases were observed decades ago when *C. saccharoperbutylacetonicum* was introduced for industrial scale fermentation in Japan[27], and twelve lytic phages affecting fermentation process with *C. saccharoperbutylacetonicum* N1-4 were described[28]. It has also been reported that the former

strain N1-4 (ATCC 13564) was lysogenized with a temperate (lysogenic) phage HM T, generating a new strain N1-4 (HMT), which could release the phage particles from the genome even without induction[29]. In addition, the N1-4 (HMT) strain can produce a phage-like bacteriocin Clostocin O with the induction by mitomycin C[2, 30]. We hypothesized that the instability of the fermentation with *C. saccharoperbutylacetonicum* might be related to the existence of prophages, and the deletion of these prophages and Clostocin O encoding sequences would improve the stability of the strain and thus enable more stable and enhanced production of the desired endproduct (BA here). The online program PHAST[31] and a manual curation were used to predict the prophage sequences in N1-4 (HMT), with four possible prophage genomes were identified: the TBP2 prophage[30] (renamed here as P1) as well as three other putative prophages which were named as P2, P3 and P4 here (Fig. 5a). The genome and description of the phage TBP2 were published in July 2020[30] and support our observations on activity and behavior of the phage, which was called in this study as P1. Besides, one additional incomplete prophage genome (without integrase gene) was found and named as P5.

In this study, all the putative prophages have been deleted to generate the set of advantageous solventogenic clostridial strains for biochemical production. We firstly constructed the mutant with single deletion of each prophage genome and generated the ΔP1, ΔP2, ΔP3 and ΔP4 strains. Because there are genes within the prophage genome possibly responsible for the normal cell metabolism, we also constructed the mutant with the deletion of only the integrase gene (without the integrase, the prophage cannot release from the chromosome), obtaining the ΔNP1, ΔNP2, ΔNP3 and ΔNP4 strains. In addition, we also constructed the mutant ΔP1234 (with the deletion of all four prophage genomes) and ΔNP1234 (with the deletion of all four integrase genes).

Fermentations were first conducted in the serum bottle to investigate the effects of the elimination of prophages on butanol production in the mutant strains. As shown in Figs. 5f, 5g & Supplementary Fig. 8, ΔP4 and ΔP1234 showed increased cell growth and butanol production, while all the other eight mutants did not demonstrate significance difference in terms of the cell growth and solvent production compared to the mother strain. ΔP4 and ΔP1234 reached the maximum OD_{600} of 17.9 and 17.6, which were 15.5% and 13.5% higher than that of the control N1-4-C strain, respectively. The butanol production in ΔP4 and ΔP1234 reached 16.8 and 17.1 g/L respectively, which were also higher than that of the control N1-4-C strain (16.0 g/L). Mutant strains with the deletion of integrases (ΔNP1, ΔNP2, ΔNP3, ΔNP4 and ΔNP1234) had no significant improvement on either cell growth or butanol production compared to the control N1-4-C (Supplementary Fig. 8).

To further study the individual prophages, we constructed the triple-deletion mutants ΔP234, ΔP134, ΔP124, ΔP123. Phage induction experiments of ΔP234, ΔP134, ΔP124, ΔP123 and ΔP1234 with mitomycin C revealed that all the mutants exhibited cell lysis (Supplementary Fig. 12). Transmission electron microscopy (TEM) results indicated that all the mutants produced the tail-like particles, which showed likely the same appearance as Clostocin O as reported previously[2] (Supplementary Fig. 6). We were not able to observe the TBP2 phage in the supernatant of ΔP234. It might be because there were too many Clostocin O particles in the view which made it difficult to observe TBP2 virions.

Based on the above results, we tentatively concluded that P5 might be responsible for the production of Clostocin O. To verify this hypothesis and obtain a more robust strain for bioproduction, P5 was deleted in ΔP234, ΔP134, ΔP124, ΔP123 and ΔP1234, obtaining ΔP2345, ΔP1345, ΔP1245, ΔP1235 and ΔP12345. The induction experiments indicated that the

cell lysis was detected in ΔP2345 with the addition of 4 μg/mL of mitomycin C at the OD_{600} of 0.2-0.5 (Supplementary Fig. 13), while no cell lysis was detected in any other mutants at any conditions with the treatment using mitomycin C. Furthermore, after induction, phage-like particles were observed only in the supernatant of ΔP2345 (Fig. 5e & Supplementary Fig. 7) suggesting that P1 is an active prophage, and, according to prediction, they were likely TBP2 virions[30]. However, the P1 phage particle image we obtained was different from that described by Schüler and co-workers[30]. The P1 particles we observed in this study consists of a head of roughly 50 nm wide and a tail of about 150 nm long (Fig. 5e & Supplementary Fig. 7); while the TBP2 phage presented in the image by Schüler *et al.*[30] comprises a 360 nm long tail as well as a base structure with tail tube and tail fibers[30]. Since the phage in this study was observed in the supernatant of ΔP2345 (only P1 remains in the genome) after induction with mitomycin C, it is more likely that the phage particles presented in Fig. 5e & Supplementary Fig. 7 are real TBP2 virions, while a different shape of TBP2 particles observed by Schüler *et al.*[30] could be result of a 'chimeric assembly' of TBP2 structural proteins with those encoded by other prophages yet presented in the host genome. Further studies will be conducted to verify this hypothesis.

After the deletion of P5, no Clostocin O particle was observed in the supernatant of ΔP12345, which confirmed that P5 indeed encoded Clostocin O. In addition, as mentioned above, no cell lysis was observed in ΔP12345 with induction (Fig. 5d & Supplementary Fig. 13), suggesting that ΔP12345 could be a more stable platform to be engineered for enhanced ester production. On the other hand, we showed above that ΔP1234 grew faster and produced more butanol than the control N1-4-C strain. Therefore, we further compared the fermentation performance of ΔP1234 vs ΔP12345 in both serum bottles and bioreactors (Figs. 5h & 5i, and Supplementary Figs. 9 & 10). Results showed that the further deletion of P5 in ΔP12345 did

not result in significant difference in cell growth or butanol production when compared to ΔP1234; actually, the butanol production in ΔP12345 was slightly lower than in ΔP1234.

We checked the cell cultures (ΔP1234, ΔP12345 vs. N1-4-C) under a microscope to exam the effects of prophage deletion on the change of cell morphology and sporulation. As shown in Supplementary Fig. 11, at 48 h of the fermentation (the lower panel of Supplementary Fig. 11), when N1-4-C demonstrated obvious cell autolysis, the majority of the cells of ΔP1234 and ΔP12345 were still in a usual rod shape with the regular sporulation.

Supplementary Note 5. Evaluation of codon-optimization of *atf1* for BA synthesis.

Different microorganisms have different codon usage preferences. The *atf1* gene was originally from *S. cerevisiae* and its genetic codon usage might not be preferable for the *C. saccharoperbutylacetonicum* host. Thus, a codon optimized *atf1* gene (designated as *atf'*) was synthesized and evaluated for potentially improved expression and BA production in the *C. saccharoperbutylacetonicum* host strain. However, fermentation results demonstrated that FJ-007 (carrying *atf1'* rather than *atf1*) actually generated slightly lower concentration of BA than FJ-004 (5.0 g/L vs. 5.5 g/L, Supplementary Table 7). The result was unexpected, but not totally surprising. Similar cases have been reported previously where the original natural gene showed better efficiency than the codon-optimized counterpart for desirable biochemical production[32]. We speculate that the natural gene might be able to transcribe into more stable mRNA structure, and thus lead to higher translation level than the codon-optimized gene[32]. In addition, it has been recently reported that codon-optimized genes could bring about toxicity to the host cells[33].

Supplementary Discussion

We firstly set out to screen the host strains and ester synthesis genes for specific ester production. With the combination of five clostridial strains (*C. saccharoperbutylacetonicum* N1-4-C, *C. pasteurianum* SD-1, *C. beijerinckii* 8052, *C. tyrobutyricum cat1::adhE1* and *cat1::adhE2*) and five ester synthesis genes (*vaat*, *saat*, *atf1*, *eht1* and *lipaseB*), we obtained very promising results. Most of the engineered strains could produce EA, BA and BB at the same time (Fig. 2), and some of the strains could also produce small amount of EB. *C. saccharoperbutylacetonicum* FJ-004 produced 5.5 g/L BA, which was the highest BA production level that has ever been reported[34]. *C. pasteurianum* J-5 produced 0.3 g/L BB, which was also significantly higher than the previously reported level of 0.05 g/L in an engineered *C. acetobutylicum* strain[34, 35]. The results confirmed our hypothesis that solventogenic clostridia are excellent platforms to be engineered for ester production.

On the other hand, we did also notice the large difference in ester production among the various clostridial host strains tested in this study. The reasons behind could include: (1) the expression (at both transcriptional and translational levels) of the five ester synthesis genes might be very different in different strains. Although all the tested strains belong to the *Clostridium* genus, their genetic backgrounds are very diverse, which might affect the gene expression and lead to the difference in specific ester formation; (2) The P_{cat} promoter from *C. tyrobutyricum* was used for the expression of all the ester synthesis genes in all strains. The expression efficiencies of these genes in various clostridial host strains could be very different, thus leading to large differences in ester production performance in different strains (especially considering that the dynamic expression of ester synthesis genes is essentially important as we demonstrated in the later section in this study); (3) the availability of precursors for ester synthesis in these various strains might be very different. Despite the similar pathways, the

availability (and especially the dynamic flux) of acetyl-CoA, butyl-CoA, acetate, butyrate, ethanol, and butanol are very different in different strains, which would lead to differences in ester production, including different capability of the stains for specific ester formation. For instance, among these strains, *C. saccharoperbutylacetonicum* produced the highest level of BA, *C. pasteurianum* produced the highest level of BB, while *C. tyrobutyricum* produced the highest level of EA (Fig. 2b).

Because the BA production in FJ-004 was significantly higher than the production levels of other esters, we decided to focus on further improving BA production in *C. saccharoperbutylacetonicum* through systematic metabolic engineering.

Butanol and acetyl-CoA are the two precursors for BA synthesis. The enhancement of the intracellular pool of these two precursors in the host could help improve BA production. We thus firstly deleted *Cspa_c47560* to save NADH and improve butanol and thus BA production. Our fermentation results showed that FJ-101 with the deletion of *Cspa_c47560* had increased BA production to 7.8 g/L. However, there was still 7.6 g/L butanol remaining at the end of fermentation with FJ-101; it was thus reasonable to speculate that the availability of acetyl-CoA was the bottleneck for further improving BA production. We tried two strategies to improve intracellular acetyl-CoA availability. One was for the enhanced regeneration of acetyl-CoA, and the other was for blocking the pathway that consumes acetyl-CoA. Comparatively, the former seemed a better strategy. By introducing a heterologous isopropanol synthesis pathway to promote the regeneration of intracellular acetyl-CoA, the FJ-301 strain could produce up to 12.9 g/L BA (Fig. 3b).

The dynamic expression of the heterologous pathway to be synchronous with the

production of the precursors could be highly beneficial for the production of the target bioproduct. On the other hand, the imbalance of intracellular metabolism and the accumulation of toxic precursors would harm the cells and lead to decreased production of the target product. Previously, synthetic regulatory tools have been developed to dynamically control the gene expression and result in enhanced production of the targeted bioproducts. For example, Zhang *et al.* developed a dynamic sensor-regulator system (DSRS) by engineering a hybrid fatty acid/acyl-CoA-regulated promoter[36]. The promoter can sense the level of the precursors for the synthesis of fatty acid ethyl ester and thus regulate the gene expression level in response to the physiological state of the cell, therefore increased the target product level. However, it needs tremendous works to screen the best responsive engineered promoter. It is well known that the intracellular metabolism especially the solvent production in clostridia is strictly regulated by the cells[37]. We hypothesized that the appropriate regulation of the BA synthesis enzyme using the native promoter of the host strain could achieve the similar effect as DSRS. In this work, four native promoters associated with BA precursors formation were selected and evaluated to control the *atf1* gene expression (Fig. 3c). Results indicated that the *atf1* gene controlled by the P_{adh} promoter showed 10.5% increase in BA production compared with the control FJ-301 strain (Fig. 3d). The P_{adh} promoter is responsible for the ethanol and butanol synthesis. The synchronous expression of alcohol dehydrogenase and ATF1 remarkably increased BA production.

Spatial organization of the enzymes associated with BA synthesis is another strategy that we employed to enhance BA production. The cross-link of the enzymes associated with BA synthesis or anchoring these enzymes onto a synthetic scaffold (PduA*) was not able to improve the BA production; while anchoring the ATF1 enzyme to the cell membrane by adding a MinD C-tag to the C-terminus of the enzyme led to significantly increased BA production.

The obtained FJ-308 produced 16.4 g/L BA, which was 20% more than that in FJ-304 (Fig. 4). The attachment of ATF1 to the cell membrane facilitated the excretion of BA from the cells, which could mitigate the intracellular toxicity caused by BA and meanwhile boost the BA synthesis.

During the fermentation, the performance of the strain for BA production was not stable, and remarkable cell lysis was also observed at the end of the fermentation. We speculated that the instability of the strain could be because of the prophages existing in the chromosome of *C. saccharoperbutylacetonicum*. Based on *in silico* analysis, we identified four putative active prophages P1-P4 as well as one inactive prophage P5 (does not have an integrase gene) in the genome of *C. saccharoperbutylacetonicum* N1-4 (HMT). In our study, P1 was found identical to the recently described lysogenic phage TBP2, while P5 was revealed to be responsible for the synthesis of Clostocin O (Fig. 5c, 5e & Supplementary Figs. 6-7). Ultimately, we obtained two mutant strains ΔP1234 and ΔP12345, both of which grew faster and produced more butanol than the wild type strain (Figs. 5f-5i; Supplementary Figs. 8-10). Thus, we further constructed the BA-producing strains FJ-1201 and FJ-1301 respectively based on ΔP1234 and ΔP12345. Fermentation results demonstrated that FJ-1201 could produce 20.3 g/L BA (Fig. 6a & Supplementary Table 1), which was the highest production level of BA that has ever been reported in any microbial biocatalyst host[34]. The BA yield in FJ-1201 reached 0.26 g/g, which was also significantly higher than the initial BA-producing strain FJ-004 (0.07 g/g). Thus, the deletion of prophages from *C. saccharoperbutylacetonicum* could not only improve the cell growth (and stability) but also increase the production of the desired endproducts (butanol or BA). These results should stimulate more attention to research on yet cryptic prophages of solventogenic clostridia, thereby helping not only to improve industrial ABE fermentation, but also to gain a deeper understanding of roles of lysogenic phages in these industrially important

bacteria. Impact of bacteriophages can be dramatic in industrial processes, and according to Jones *et al.*[38] - the growth rate of the lysogenic strain was found to be slower than the non-lysogenic parent strain and exhibited reduced solvent production. Furthermore, the solvent yield can cut by half for almost a year due to the presence of phages in bioprocessing environments[39].

In addition, we also noticed that the BB production in FJ-1201 reached 0.9 g/L, which was significantly higher than that in *C. pasteurianum* J-5 (0.3 g/L, the highest BB production level based on our initial screening of the strains and enzymes for ester production) (Fig. 6a & Supplementary Table 1). We further overexpressed *saat* (instead of *atf1*) in the strain, and obtained the FJ-1202 strain in which the BB production reached 1.3 g/L (Fig. 6d).

Both the BA-producing *C. saccharoperbutylacetonicum* FJ-1201 and the BB-producing *C. saccharoperbutylacetonicum* FJ-1202 performed well when biomass hydrolysates were used as the substrate for the fermentation. FJ-1201 could produce 17.8 g/L BA and FJ-1202 could produce 0.9 g/L BB from biomass hydrolysates (with no need to supplement any exogenous nitrogen source). Although these levels were slightly lower than when glucose was used as the substrate for the fermentation with the same strain, the operation eliminated the requirement of yeast extract and tryptone and thus would significantly decrease the cost of the bioprocess for fatty acid ester production.

Supplementary Table 1. Butyl acetate production in the mutant strains with prophage deleted from the chromosome.*

	**FJ-1201 48 h**	**FJ-1201 72 h**	**FJ-1301 48 h**	**FJ-1301 72 h**
**EA**	< 0.01	< 0.01	< 0.01	< 0.01
**BA**	19.3 ± 0.5	19.7 ± 1.7	19.1 ± 0.5	19.4 ± 0.7
**BB**	0.9 ± 0.1	0.7 ± 0.1	0.8 ± 0.0	0.7 ± 0.0
**BA (aqueous phase)**	0.6 ± 0.1	0.6 ± 0.1	0.5 ± 0.0	0.5 ± 0.1

*EA: ethyl acetate; BA: butyl acetate; BB: butyl butyrate. All values are in g/L. Source data are provided as a Source Data file.

Supplementary Table 2. Ester production in FJ-1201 using biomass hydrolysates as the substrate with or without supplementation of exogenous organic nitrogen source.*

	**0Y+0T#**	**1Y+3T**	**2Y+6T**
**EA**	< 0.01	< 0.01	< 0.01
**BA**	17.5 ± 0.2	16.9 ± 0.5	16.2 ± 0.7
**BB**	0.1 ± 0.0	0.2 ± 0.1	0.2 ± 0.1
**BA (aqueous phase)**	0.3 ± 0.0	0.2 ± 0.0	0.2 ± 0.0

*EA: ethyl acetate; BA: butyl acetate; BB: butyl butyrate. All values are in g/L.
#Y=Yeast, T=Typtone; 0Y+0T: 0 g/L Y and 0 g/L T; 1Y+3T: 1 g/L Y and 3 g/L T; 2Y+6T: 2 g/L Y and 6 g/L T.
Source data are provided as a Source Data file.

Supplementary Table 3. Annual and unit costs for the annual production of 83,332 metric tonne (MT) of butyl acetate (BA) from corn stover.

Description	Annual Use	Annual cost	Unit BA cost
Raw materials (MT)			
Corn stover	875,000	45,045,000	
Sodium hydroxide	29,343	3,403,788	
Celullase (MT)	4,575	19,398,000	
Hexadecane	185	740,000	
Sulfuric acid	36,107	1,476,697	
WWT nutrients*	422	190,322	
Boiler chemicals	4	31,547	
Cooling tower chemicals	17	61,697	
Subtotal raw materials		70,347,051	844.2
Utilities (MT)			
Water	4,501,681	990,370	
Natural gas	71,383	13,205,855	
Subtotal utilities		14,196,225	170.4
Labor			
Total salaries		2,500,000	
Labor burden		2,250,000	
Subtotal labor		4,750,000	57.0
Other overhead			
Maintenance		1,628,312	
Property insurance		1,808,111	
Subtotal overhead		3,436,422	41.2
Capital depreciation		20,955,052	251.5
Co-product credit			
Butanol (MT)	9,673	-8,705,700	
Isopropanol (MT)	13,555	-15,528,250	
Surplus electricity (Mwh)**	111,104	-7,221,732	
Subtotal co-product credit		-31,515,682	-378.2
Net operating cost		82,169,068	986.0

*WWT: Wastewater treatment; **Mwh: Megawatt hour.

Supplementary Table 4. Key operation parameters for the corn stover conversion and butyl acetate fermentation considered in the techno-economic analysis (TEA).

**Pretreatment and hydrolysis**	
Pretreatment solids loading	25 wt%
Sodium hydroxide loading	40 kg/MT dry corn stover
Deacetylation incubation temperature	80 °C
Deacetylation incubation time	2 hours
Disk milling energy consumption	212 kWh/MT dry corn stover
Enzymatic hydrolysis solids loading	20 wt%
Enzyme loading	19 mg protein/g cellulose
Enzymatic hydrolysis temperature	48 °C
Enzymatic hydrolysis time	84 hours
Cellulose hydrolysis efficiency	82%
Hemicellulose hydrolysis efficiency	74%
**Fermentation**	
Initial sugar concentration	72.9 g/L
Hexadecane load	1:1 by volume
Fermentation temperature	30 °C
Fermentation time	96 hours
Butyl acetate yield	0.25 g/g consumed sugar
Butanol yield	0.03 g butanol/g consumed sugar
Isopropanol yield	0.04 g isopropanol/g consumed sugar
Additional nutrients	None (based on verified experiments)

Supplementary Table 5. Summary of key raw material costs.

Item	Cost ($)
**Raw materials and utilities**	
Corn stover (20% moisture)	51.5/MT [a]
Sodium hydroxide	116/MT [b]
Cellulase	4,240/MT [a]
Hexadecane	4,000/MT [b]
Wastewater treatment chemicals	4,51/MT [b]
Boiler chemicals	8,336/MT [a]
Cooling tower chemicals	3,668/MT [a]
Sulfuric acid	41.0/MT [b]
Freshwater	0.22/MT [a]
Natural gas	185/MT [b]
**Co-product credits**	
Butanol	900/MT [b]
Isopropanol	1150/MT [b]
Surplus electricity	0.065/kWh [b]
**Fixed operating costs**	
Labor costs	2,500,000 [c]
Labor burden	90% of labor cost
Maintenance	3% of ISBL[d]
Property insurance	0.7% of fixed capital investment

[a]The price of corn stover and other chemicals were from the previous literature including Humbird *et al.* [4], Chen *et al.*[3], and Dalle Ave and Adams[40]; MT: metric tonne.
[b]Data from different sources, including the ICIS chemical price report and industrial quotes.
[c]Assuming 50 employees with an average annual salary of $50,000 per employee.
[d]Inside Battery Limits.

Supplementary Table 6. Project total capital investment ($ million) for the processes.

	Purchased cost ($ million)	Installed cost ($ million)
Feedstock handling	15.5	26.4
Pretreatment & hydrolysis	18.0	29.8
Fermentation	36.6	55.2
Production recovery & upgrading	6.6	12.6
Product & chemical Storage	1.3	2.2
Wastewater treatment	59.8	59.8
Electricity & steam generation	34.4	68.4
Utilities	4.5	8.3
**Total installed equipment cost**		**262.7**
Other direct cost		17.1
Total direct cost (TDC)		279.8
Total indirect costs (TIC)		167.9
**Fixed capital investment (FCI)**		**447.6**
Land		1.8
Working capital		22.4
**Total capital investment (TCI)**		**471.8**

Supplementary Table 7. Butyl acetate production in the engineered strains for the evaluation of the effect of gene codon optimization.*

	**FJ-004**	**FJ-007**
**EA**	< 0.01	< 0.01
**BA**	5.5 ± 0.5	5.0 ± 0.2
**BB**	0.01 ± 0.00	< 0.01
**Glucose consumption**	79.3 ± 0.1	76.7 ± 2.4
**Lactate**	0.0 ± 0.0	0.7 ± 0.1
**Acetate**	0.3 ± 0.0	0.2 ± 0.1
**Ethanol**	1.0 ± 0.0	1.0 ± 0.0
**Acetone**	5.0 ± 0.2	3.6 ± 0.2
**Butyrate**	0.1 ± 0.0	0.0 ± 0.0
**Butanol**	7.8 ± 0.0	9.5 ± 0.1

* EA: ethyl acetate; BA: butyl acetate; BB: butyl butyrate. All values are in g/L. Source data are provided as a Source Data file.

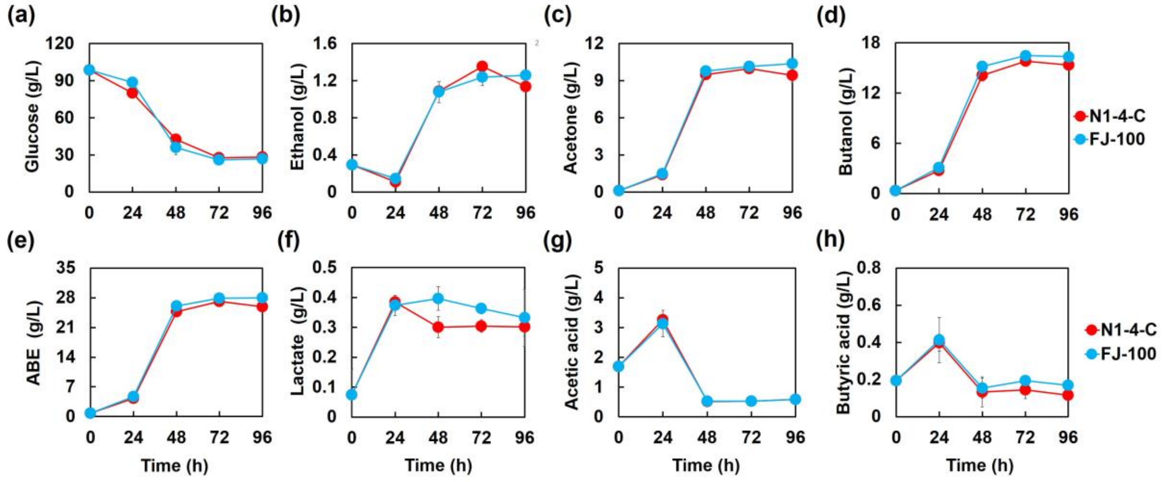

Supplementary Fig. 1. Fermentation results in serum bottles with *Clostridium saccharoperbutylacetonicum* N1-4-C and the FJ-100 strain. (a) Glucose consumption; (b) Ethanol; (c) Acetone; (d) Butanol; (e) Total ABE; (f) Lactate; (g) Acetic acid; (h) Butyric acid. Data are presented as mean values +/- SD (n = 3). Source data are provided as a Source Data file.

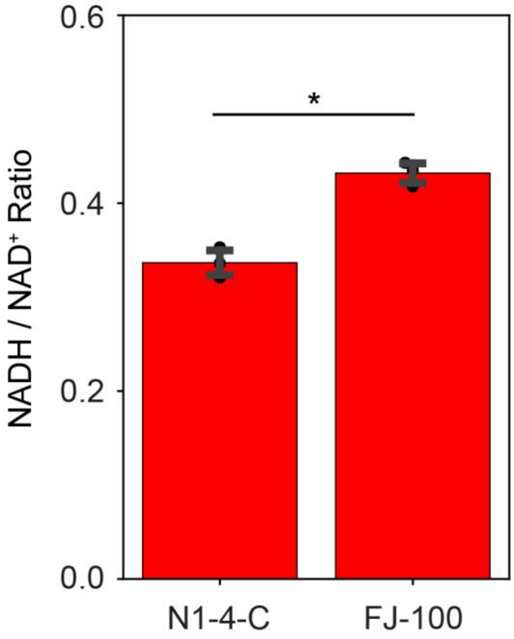

Supplementary Fig. 2. Comparison of the intracellular NADH/NAD^+ ratio between FJ-100 (in which *Cspa_c47560* was deleted) and N1-4-C (mother strain). Data are presented as mean values +/- SD (n = 3). The asterisk indicates a statistically significant difference based on Two-Sample t-Test ($p = 0.0013$). Source data are provided as a Source Data file.

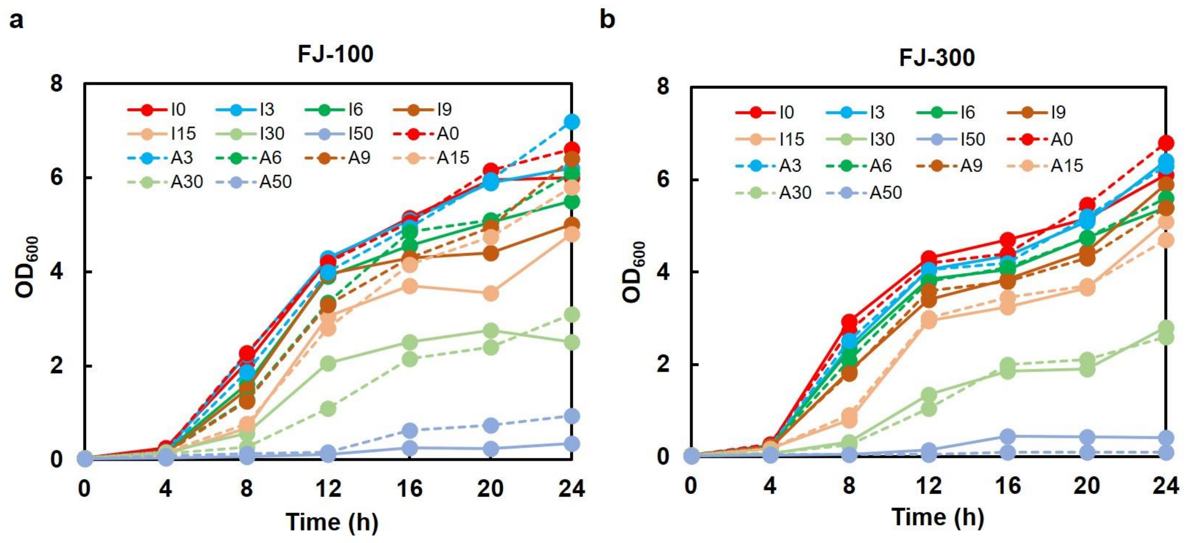

Supplementary Fig. 3. Comparison of the relative inhibition of isopropanol vs. acetone on cell growth of (a) FJ-100 and (b) FJ-300. In the figure legend, 'I' represents 'isopropanol', and 'A' represents 'acetone'. The number following the letter stands for the concentration (in g/L) of 'I' ('isopropanol') or 'A' ('acetone') supplemented in that specific culture. For example, 'I3' means that 3 g/L of isopropanol was supplemented into that cell culture. The data are shown as the mean of two independent biological replicates (n = 2). Source data are provided as a Source Data file.

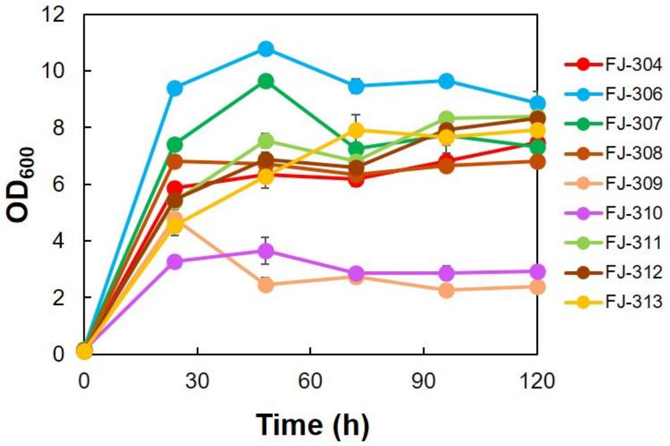

Supplementary Fig. 4. Cell growth profiles of the various butyl acetate-producing mutants. Data are presented as mean values +/- SD (n = 3). Source data are provided as a Source Data file.

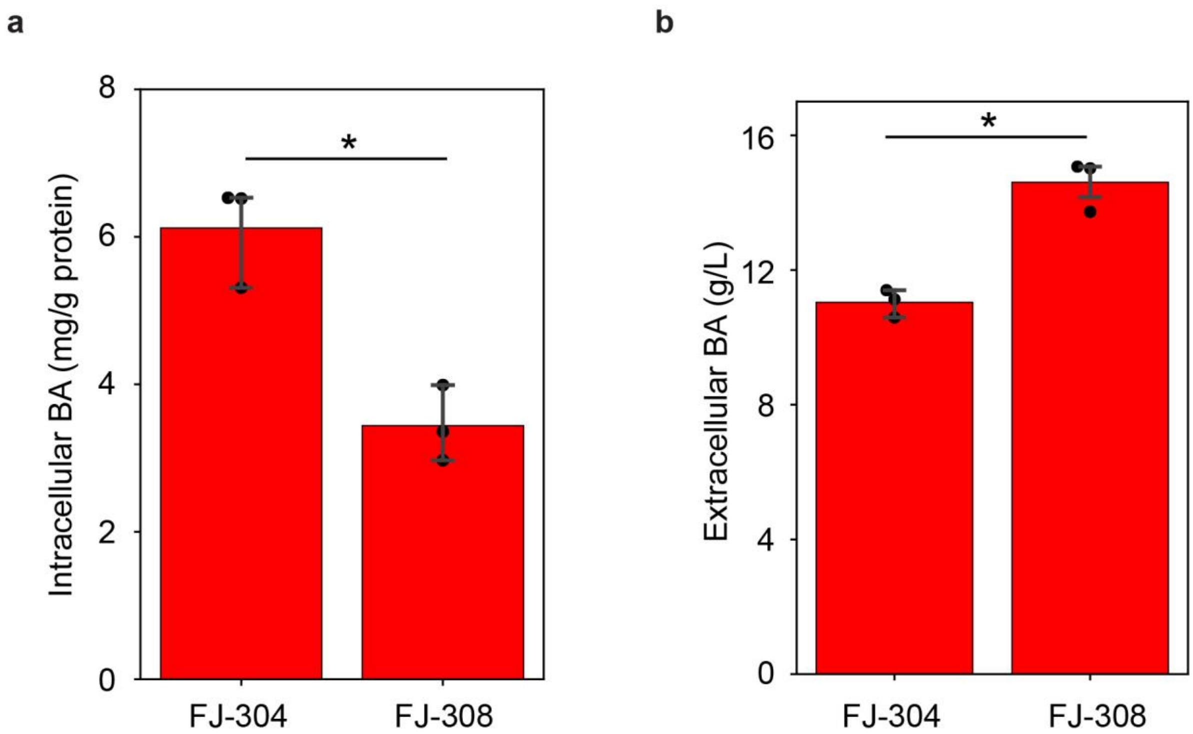

Supplementary Fig. 5. The concentration of intracellular butyl acetate (BA) (a) and extracellular BA (b) in FJ-304 and FJ-308. The samples were taken at 60 h of the fermentation. The concentration of intracellular BA was normalized against the total amount of protein in the sample. Data are presented as mean values +/- SD (n = 3). The asterisk indicates a statistically significant difference based on Two-Sample t-Test ($p = 0.0059$ for (a) and $p = 0.0020$ for (b)). Source data are provided as a Source Data file.

(a) (b)

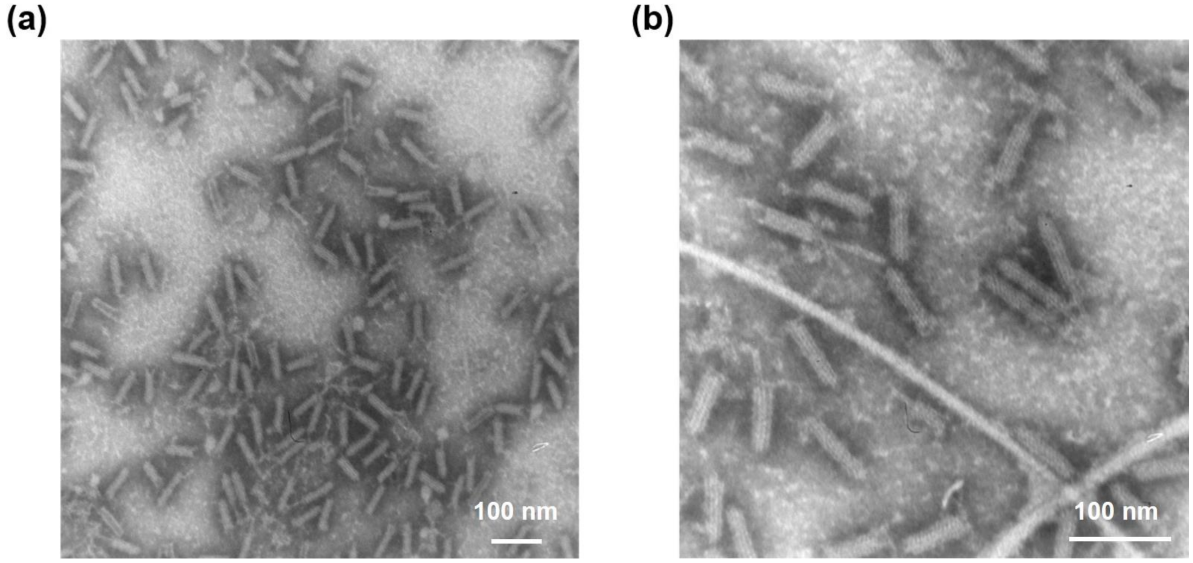

Supplementary Fig. 6. Transmission electron microscopy (TEM) picture of Clostocin O. The magnification of (a) is 100x, while the magnification of (b) is 200x. Similar results were observed in six independent experiments. Source data are provided as a Source Data file.

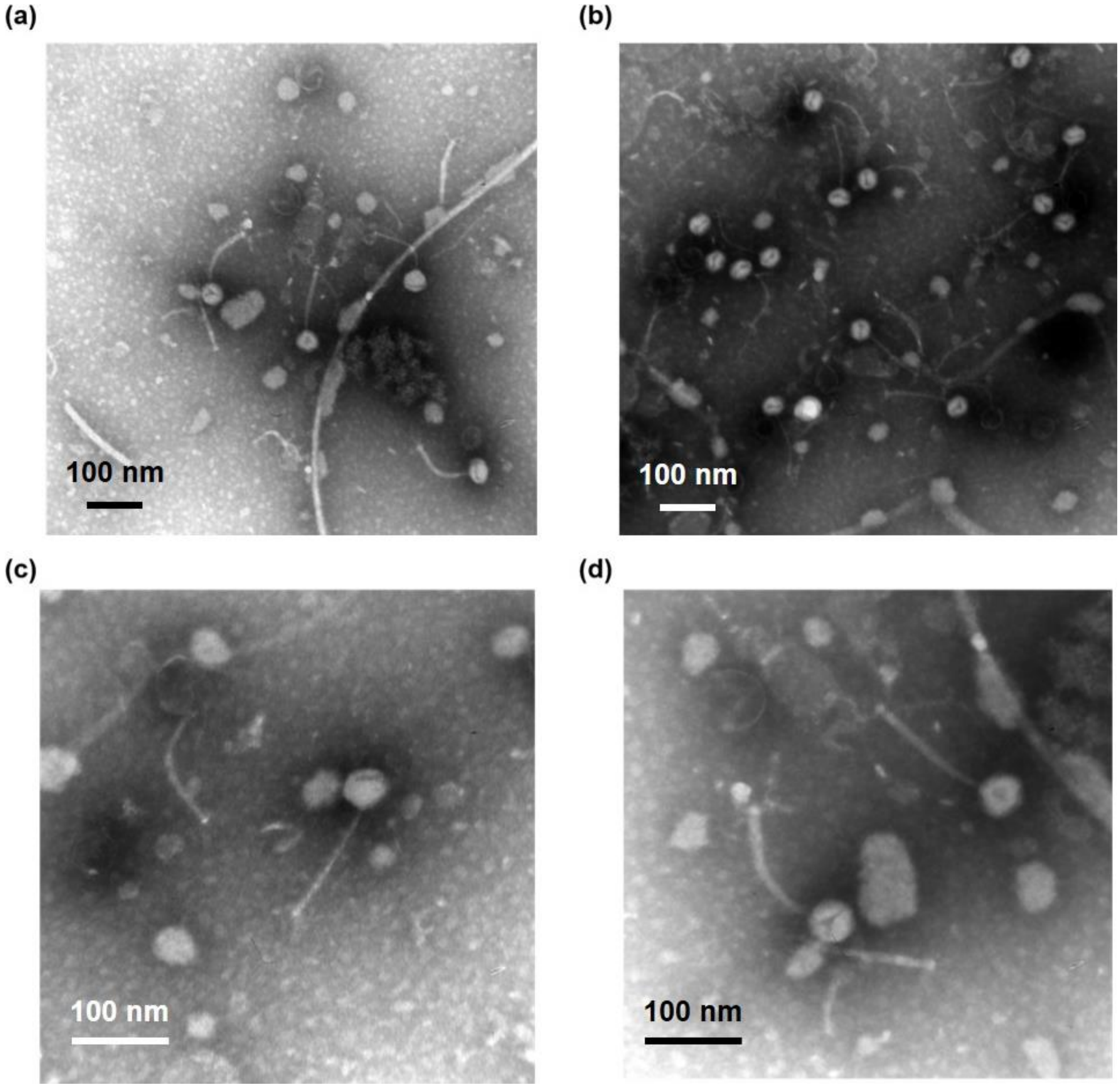

Supplementary Fig. 7. TBP2 phage observed in the ΔP2345 strain upon induction. The magnification of (a, b) is 100x, while the magnification of (c, d) is 200x. Similar results were observed in two independent experiments. Source data are provided as a Source Data file.

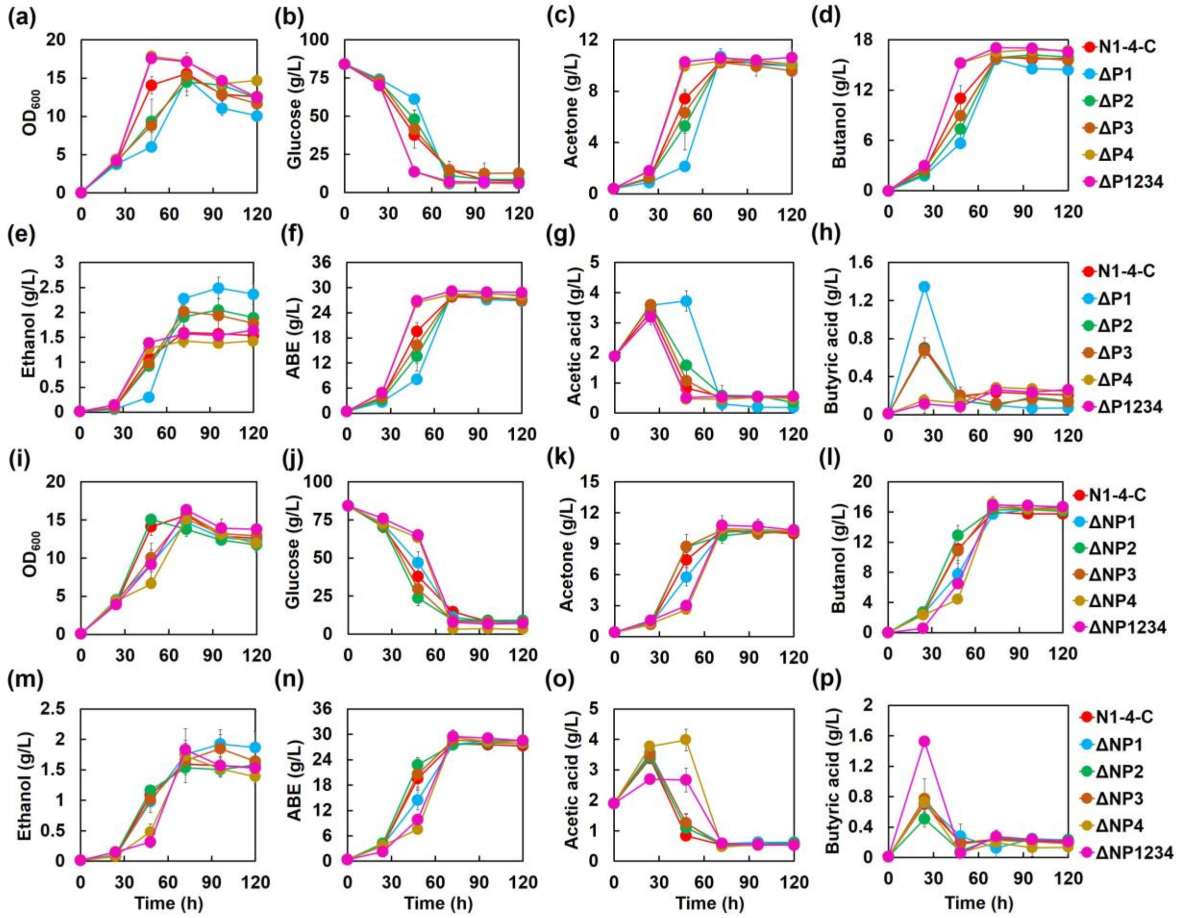

Supplementary Fig. 8. Fermentation results in serum bottles with *Clostridium saccharoperbutylacetonicum* N1-4-C and the mutant strains with prophage deleted. (a-h): Acetone-Butanol-Ethanol (ABE) fermentation results for ΔP1, ΔP2, ΔP3, ΔP4, ΔP1234 as compared to the control N1-4-C. (i-p): ABE fermentation results for ΔNP1, ΔNP2, ΔNP3, ΔNP4, ΔNP1234 as compared to the control N1-4-C. Data are presented as mean values +/- SD (n = 3). Source data are provided as a Source Data file.

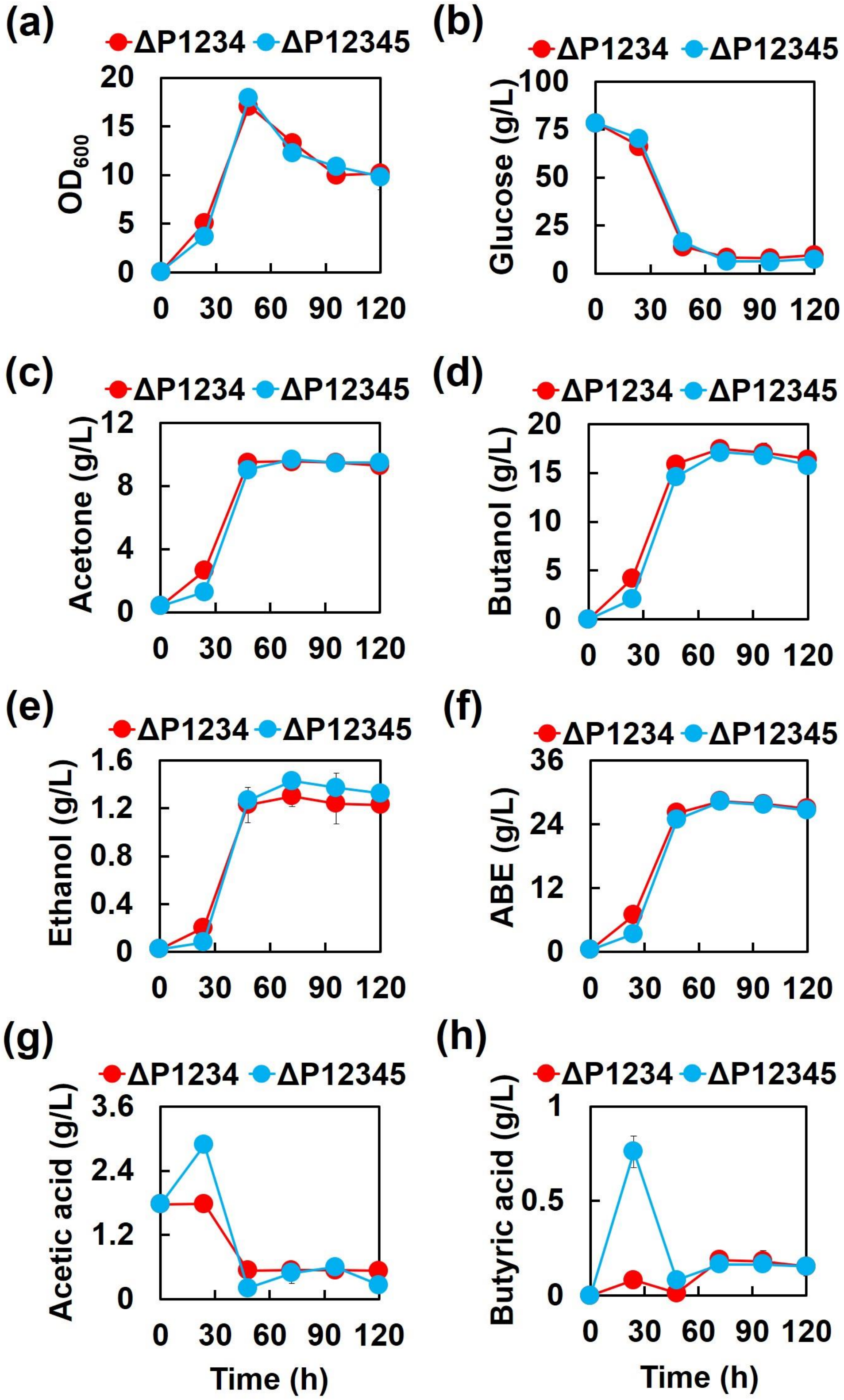

Supplementary Fig. 9. Acetone-Butanol-Ethanol (ABE) fermentation results in serum bottles with *Clostridium saccharoperbutylacetonicum* ΔP1234 and ΔP12345. (a) OD_{600}; (b) Glucose consumption; (c) Acetone; (d) Butanol; (e) Ethanol; (f) Total ABE; (g) Acetic acid; (h) Butyric acid. Data are presented as mean values +/- SD (n = 3). Source data are provided as a Source Data file.

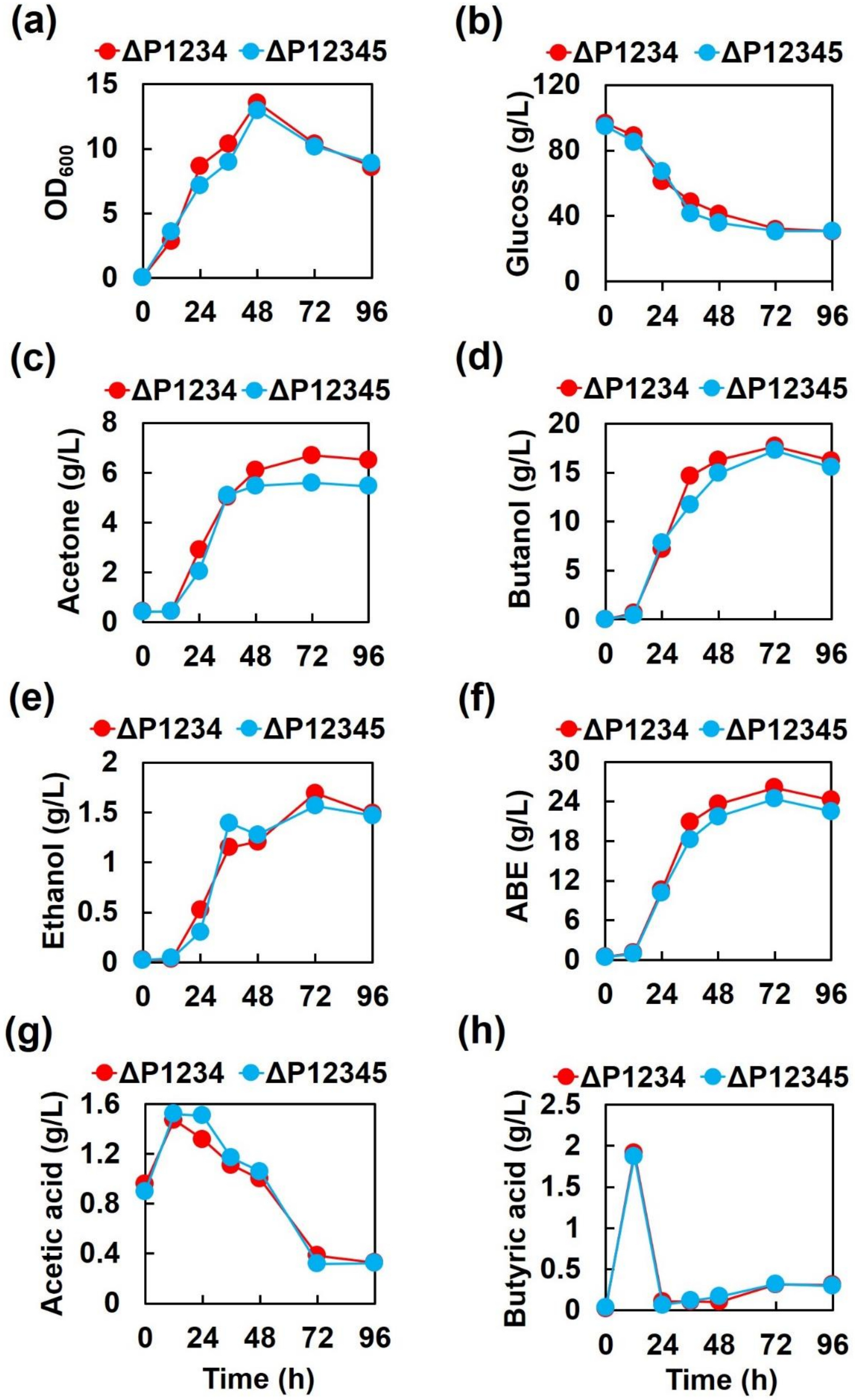

Supplementary Fig. 10. Acetone-Butanol-Ethanol (ABE) fermentation results in 500-mL bioreactors with *Clostridium saccharoperbutylacetonicum* ΔP1234 and ΔP12345. (a) OD_{600}; (b) Glucose; (c) Acetone; (d) Butanol; (e) Ethanol; (f) ABE; (g) Acetic acid; (h) Butyric acid. Source data are provided as a Source Data file.

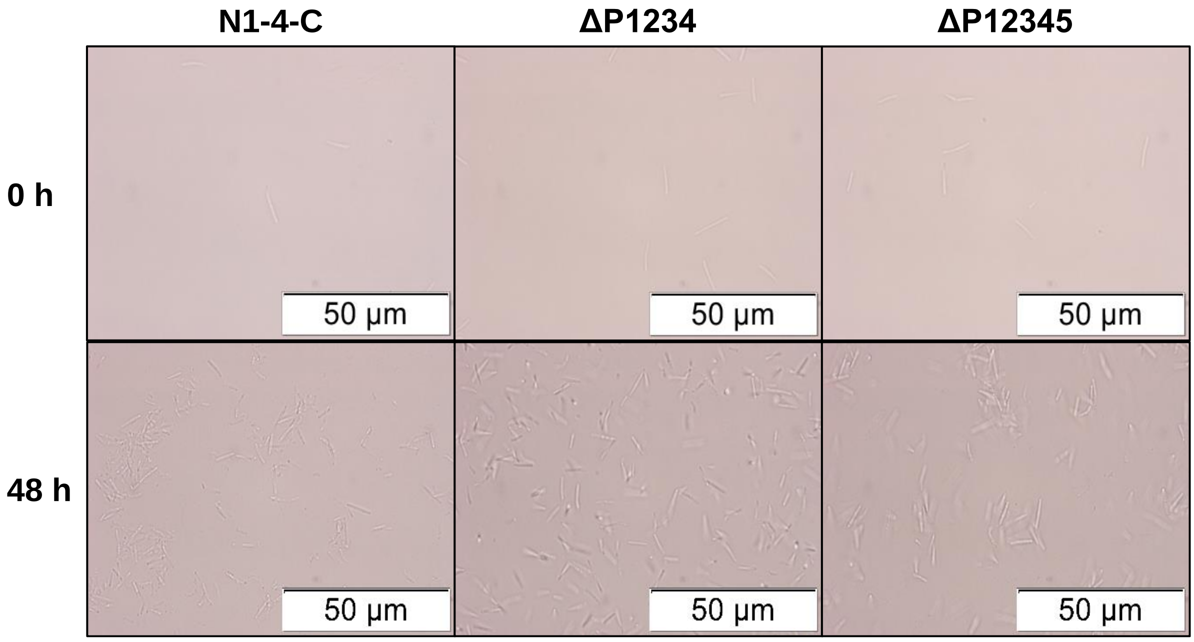

Supplementary Fig. 11. The cell morphology of the prophage-deleted mutant strains (ΔP1234 and ΔP12345) vs. the mother strain (N1-4-C) grown under fermentation conditions. Images were taken using an Olympus BX53F Upright Microscope (phase contrast mode) equipped with an Olympus DP73 Camera (Olympus Corporation, Shinjuku-ku, Japan) (100x). Similar results were observed in three independent experiments. Source data are provided as a Source Data file.

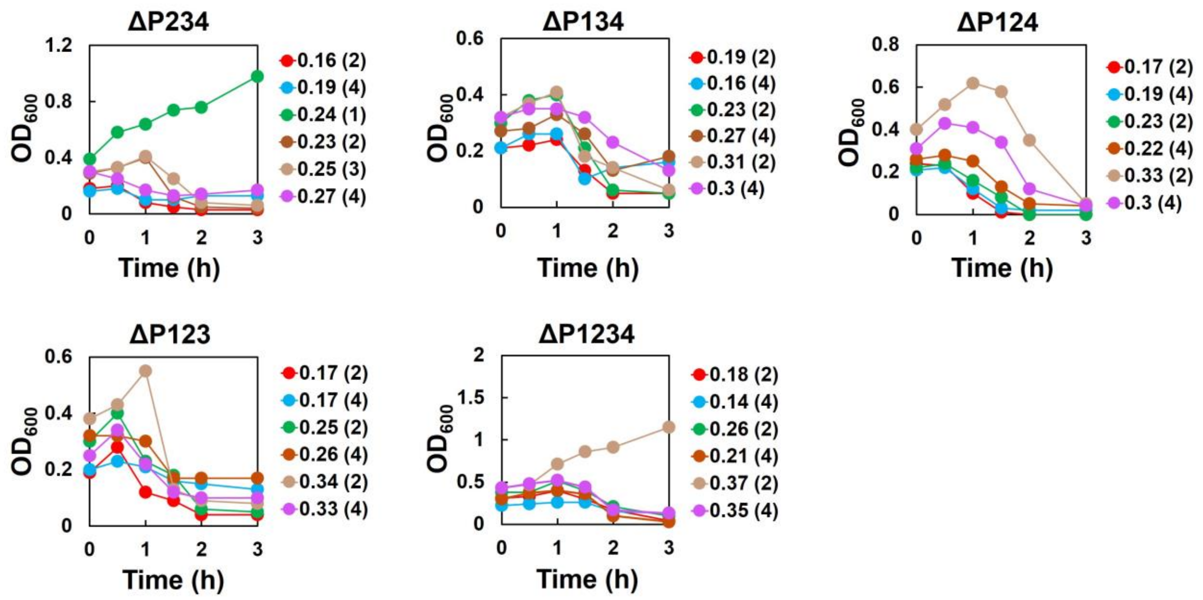

Supplementary Fig. 12. Cell lysis in various prophage deletion mutants upon induction with different concentrations of mitomycin C. The value on the right side of the cell growth profile figure represents the actual OD_{600} value at which mitomycin C (with the applied concentration included in the parentheses, μg/ml) was added for the induction. Source data are provided as a Source Data file.

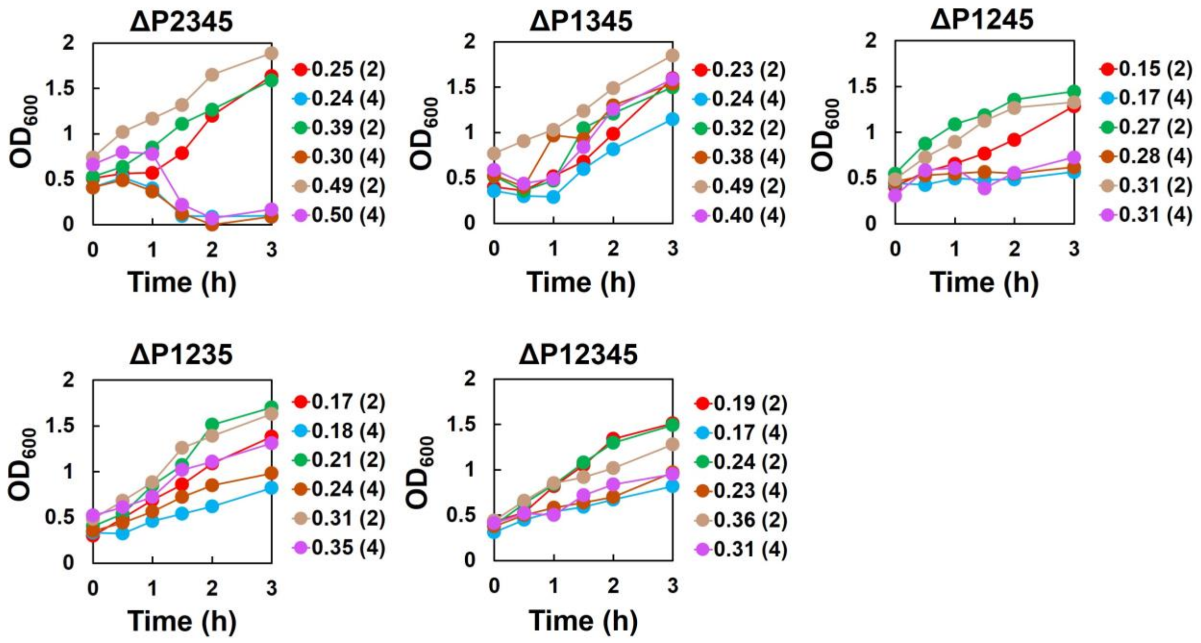

Supplementary Fig. 13. Cell lysis in various prophage deletion mutants upon induction with different concentrations of mitomycin C. The value on the right side of the cell growth profile figure represents the actual OD_{600} value at which mitomycin C (with the applied concentration included in the parentheses, μg/ml) was added for the induction. Source data are provided as a Source Data file.

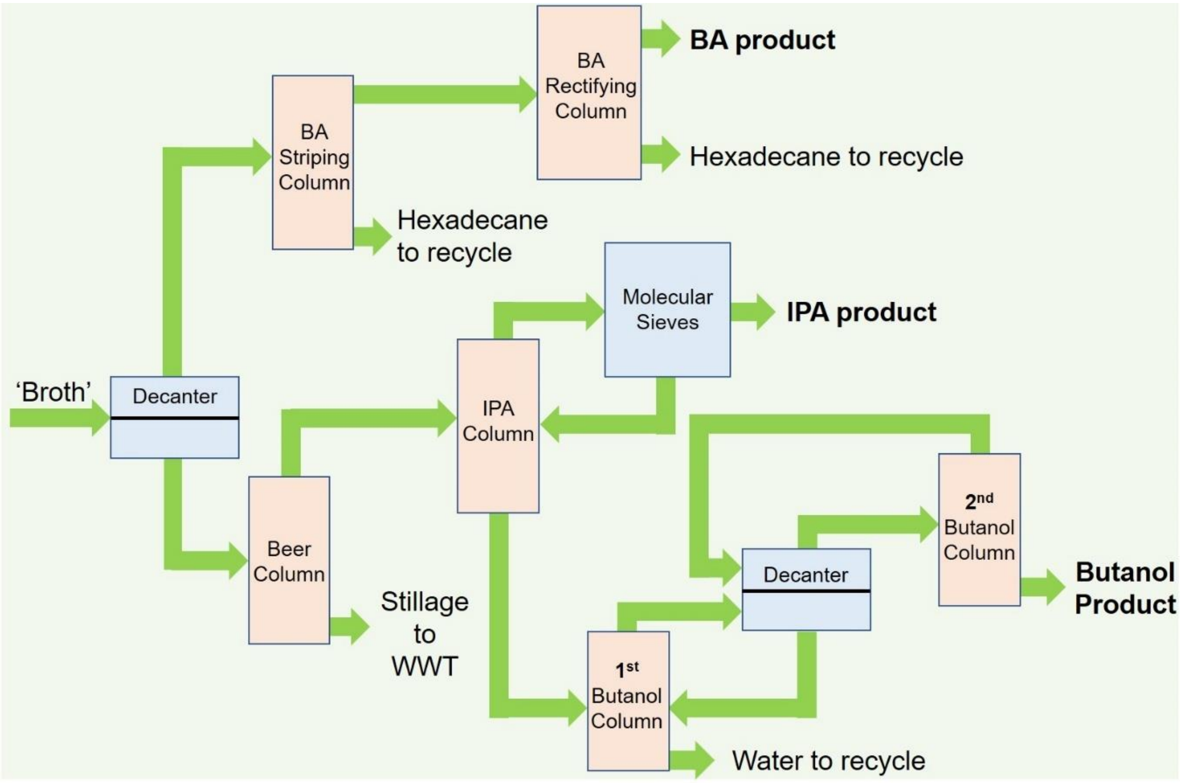

Supplementary Fig. 14. Process flow diagram of product recovery for the techno-economic analysis (TEA). BA: butyl acetate; IPA: isopropanol; WWT: Wastewater treatment.

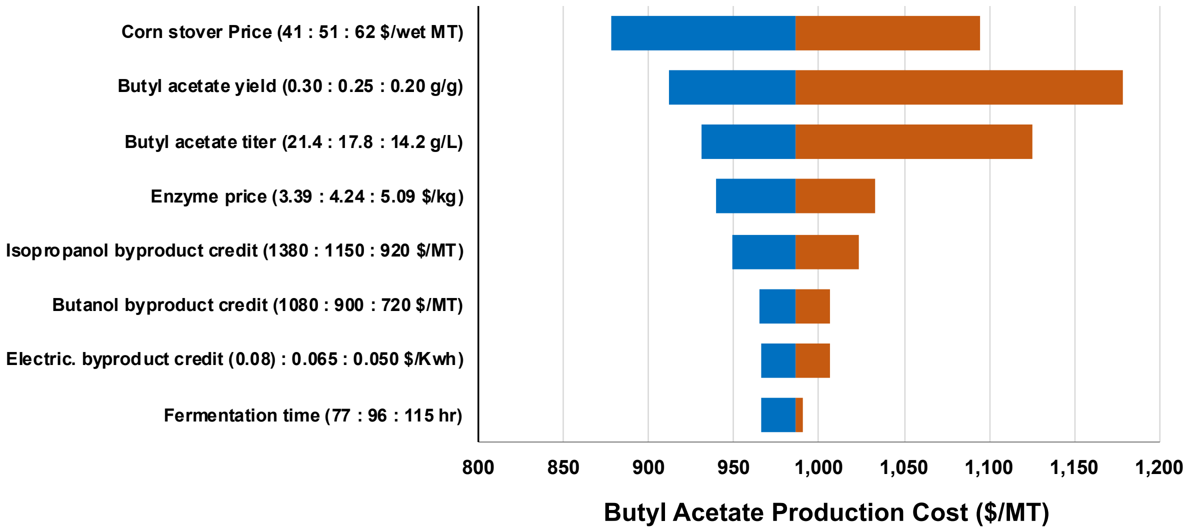

Supplementary Fig. 15. Sensitivity of butyl acetate production cost to different parameters. The numbers in brackets in Y-axis are the potential low, base and high values of each parameter. Source data are provided as a Source Data file.

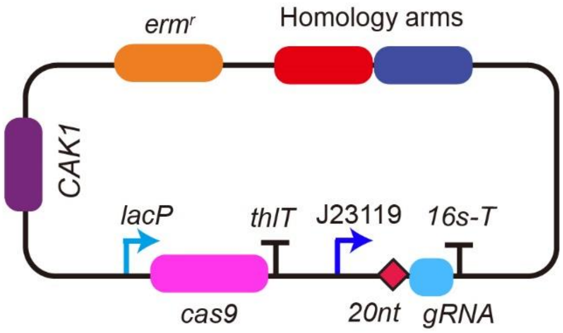

Supplementary Fig. 16. Diagram of the CRISPR-Cas9 plasmid for gene deletion. The plasmid for gene deletion was constructed based on pYW34[18, 21]. The expression of *cas9* was controlled by a lactose inducible promoter *lacP*. The expression of gRNA was controlled by the J23119 synthetic promoter. Two homology arms were inserted into the plasmid. *CAK1*: *Clostridium beijerinckii* Gram-positive replicon; *erm*^{r}: the erythromycin resistant marker; *thlT*: terminator of the thiolase gene (*Cbei_0411*); 16s-T: terminator of the 16s rDNA gene.

Supplementary references

1. Sedmak, J.J., Grossberg, S. E. A rapid, sensitive, and versatile assay for protein using Coomassie brilliant blue G250. *Analytical Biochemistry* **79**, 544-552 (1977).
2. Ogata, S., Mihara, O., Ikeda, Y. & Hongo, M. Inducible phage tail-like particles of *Clostridium saccharoperbutylacetonicum* and its related strains. *Agricultural and Biological Chemistry* **36**, 1413-1421 (1972).
3. Chen, X. et al. Techno-economic analysis of the deacetylation and disk refining process: characterizing the effect of refining energy and enzyme usage on minimum sugar selling price and minimum ethanol selling price. *Biotechnol Biofuels* **8**, 173 (2015).
4. D. Humbird, R.D., L. Tao, C. Kinchin,, D. Hsu, A.A., P. Schoen, J. Lukas, B. Olthof, M. Worley, & D. Sexton, a.D.D. Process design and economics for biochemical conversion of lignocellulosic biomass to ethanol:dilute-acid pretreatment and enzymatic hydrolysis of corn stover. (No. NREL/TP-5100-47764). National Renewable Energy Lab.(NREL), Golden, CO (United States). (2011).
5. Baral, N.R. & Shah, A. Techno-economic analysis of cellulose dissolving ionic liquid pretreatment of lignocellulosic biomass for fermentable sugars production. *Biofuels, Bioproducts and Biorefining* **10**, 70-88 (2016).
6. Luyben, W. Control of the heterogeneous azeotropic n-Butanol/Water distillation system. *Energy & Fuels* **22**, 4249-4258 (2008).
7. Tao, L., He, X., Tan, E.C.D., Zhang, M. & Aden, A. Comparative techno-economic analysis and reviews of n-butanol production from corn grain and corn stover. *Biofuels, Bioproducts and Biorefining* **8**, 342-361 (2014).
8. Cho J, J.J. Optimization study on the azeotropic distillation process for isopropyl alcohol dehydration. *Korean Journal of Chemical Engineering* **23**, 1-7 (2006).
9. Lide, D.R. CRC handbook of chemistry and physics (85th edition), Section 6. Fluid Properties., (CRC press, Florida, USA, 2005).
10. Doherty, M.F., Malone, M. F. Conceptual design of distillation systems. (McGraw-Hill, New York, USA, 2001).
11. Huang, H., Long, S. & Singh, V. Techno-economic analysis of biodiesel and ethanol co-production from lipid-producing sugarcane. *Biofuels, Bioproducts and Biorefining* **10**, 299-315 (2016).
12. Chemical engineering magazine plant cost index. *Chemical Engineering Magazine*, https://www.chemengonline.com/site/plant-cost-index/ (2020).
13. Max, S.P., Klaus, D.T., Ronald, E.W Plant design and economics for chemical engineers. (McGraw-Hill 2003).
14. Haas, M.J., McAloon, A.J., Yee, W.C. & Foglia, T.A. A process model to estimate biodiesel production costs. *Bioresource Technology* **97**, 671-678 (2006).
15. Tao, L. & Aden, A. The economics of current and future biofuels. *In Vitro Cellular & Developmental Biology - Plant* **45**, 199-217 (2009).
16. Pyne, M.E., Moo-Young, M., Chung, D.A. & Chou, C.P. Development of an electrotransformation protocol for genetic manipulation of *Clostridium pasteurianum*. *Biotechnology for Biofuels* **6**, 50 (2013).
17. Pyne, M.E., Bruder, M.R., Moo-Young, M., Chung, D.A. & Chou, C.P. Harnessing

heterologous and endogenous CRISPR-Cas machineries for efficient markerless genome editing in *Clostridium*. *Scientific Reports* **6**, 25666 (2016).
18. Wang, Y. et al. Bacterial genome editing with CRISPR-Cas9: deletion, Integration, single nucleotide modification, and desirable "clean" mutant selection in *Clostridium beijerinckii* as an example. *ACS Synthetic Biology* **5**, 721-732 (2016).
19. Zhang, J., Zong, W., Hong, W., Zhang, Z.-T. & Wang, Y. Exploiting endogenous CRISPR-Cas system for multiplex genome editing in *Clostridium tyrobutyricum* and engineer the strain for high-level butanol production. *Metabolic Engineering* **47**, 49-59 (2018).
20. Herman, N.A. et al. Development of a High-Efficiency Transformation Method and Implementation of Rational Metabolic Engineering for the Industrial Butanol Hyperproducer Clostridium saccharoperbutylacetonicum Strain N1-4. *Applied and environmental microbiology* **83**, e02942-02916 (2017).
21. Wang, S., Dong, S., Wang, P., Tao, Y. & Wang, Y. Genome editing in *Clostridium saccharoperbutylacetonicum* N1-4 with the CRISPR-Cas9 system. *Applied and Environmental Microbiology* **83**, e00233-00217 (2017).
22. Wang, S., Huang, H., Moll, J. & Thauer, R.K. NADP+ reduction with reduced ferredoxin and NADP+ reduction with NADH are coupled via an electron-bifurcating enzyme complex in *Clostridium kluyveri*. *Journal of Bacteriology* **192**, 5115-5123 (2010).
23. Liang, J., Huang, H. & Wang, S. Distribution, evolution, catalytic mechanism, and physiological functions of the flavin-based electron-bifurcating NADH-dependent reduced ferredoxin: NADP(+) oxidoreductase. *Frontiers in Microbiology* **10**, 373 (2019).
24. Wang, S., Huang, Haiyan, Kahnt, Jörg, Thauer, Rudolf K. A reversible electron-bifurcating ferredoxin- and NAD-dependent [FeFe]-hydrogenase (HydABC) in *Moorella thermoacetica*. *Journal of Bacteriology* **195**, 1267-1275 (2013).
25. Wang, S., Huang, H., Kahnt, J. & Thauer, R.K. *Clostridium acidurici* electron-bifurcating formate dehydrogenase. *Applied and Environmental Microbiology* **79**, 6176-6179 (2013).
26. Wang, S. et al. NADP-specific electron-bifurcating [FeFe]-hydrogenase in a functional complex with formate dehydrogenase in *Clostridium autoethanogenum* grown on CO. *Journal of Bacteriology* **195**, 4373-4386 (2013).
27. Hongo, M. et al. Butanol fermentation. XXXII. Abnormal fermentations in acetone-butanol production on a plant scale by use of *Clostridium saccharoperbutylacetonicum*. *Journal of Agricultural and Chemistry* **39**, 252-256 (1965).
28. Hongo, M., Aono, T. & Murata, A. Bacteriophages of *Clostridium saccharoperbutylacetonicum* Part IV. Serological Characteristics of the Twelve HM-Phages. *Agricultural and Biological Chemistry* **30**, 399-405 (1966).
29. Hongo, M., Murata, A. & Ogata, S. Bacteriophages of *Clostridium saccharoperbutylacetonicum*: Part XVI. Isolation and some characters of a temperate phage. *Agricultural and Biological Chemistry* **33**, 337-342 (1969).
30. Schuler, M.A., Stegmann, B.A., Poehlein, A., Daniel, R. & Durre, P. Genome sequence analysis of the temperate bacteriophage TBP2 of the solvent producer *Clostridium*

saccharoperbutylacetonicum N1-4 (HMT, ATCC 27021). *FEMS Microbiology Letters* **367**, fnaa103 (2020).
31. Zhou, Y., Liang, Y., Lynch, K.H., Dennis, J.J. & Wishart, D.S. PHAST: a fast phage search tool. *Nucleic Acids Research* **39**, W347-W352 (2011).
32. Feng, J. et al. Enhancing poly-γ-glutamic acid production in *Bacillus amyloliquefaciens* by introducing the glutamate synthesis features from *Corynebacterium glutamicum*. *Microbial Cell Factories* **16**, 88 (2017).
33. Mittal, P., Brindle, J., Stephen, J., Plotkin, J.B. & Kudla, G. Codon usage influences fitness through RNA toxicity. *Proceedings of the National Academy of Sciences* **115**, 8639-8644 (2018).
34. Noh, H.J., Woo, J.E., Lee, S.Y. & Jang, Y.-S. Metabolic engineering of *Clostridium acetobutylicum* for the production of butyl butyrate. *Applied Microbiology and Biotechnology* **102**, 8319-8327 (2018).
35. Noh, H.J., Lee, S.Y. & Jang, Y.-S. Microbial production of butyl butyrate, a flavor and fragrance compound. *Applied Microbiology and Biotechnology* **103**, 2079-2086 (2019).
36. Zhang, F., Carothers, J.M. & Keasling, J.D. Design of a dynamic sensor-regulator system for production of chemicals and fuels derived from fatty acids. *Nature Biotechnology* **30**, 354 (2012).
37. Kosaka, T., Nakayama, S., Nakaya, K., Yoshino, S. & Furukawa, K. Characterization of the *sol* operon in butanol-hyperproducing *Clostridium saccharoperbutylacetonicum* strain N1-4 and its degeneration mechanism. *Bioscience, Biotechnology, and Biochemistry* **71**, 58-68 (2007).
38. David T. Jones, Matt Shirley, Xiyang Wu & Keis, S. Bacteriophage Infections in the Industrial Acetone Butanol (AB) Fermentation Process. *Journal of Molecular Microbiology and Biotechnology*, 21-26 (2000).
39. Sun, W.-J. et al. A novel bacteriophage KSL-1 of 2-Keto-gluconic acid producer *Pseudomonas fluorescens* K1005: isolation, characterization and its remedial action. *BMC microbiology* **12**, 1-8 (2012).
40. Dalle Ave, G. & Adams, T.A. Techno-economic comparison of Acetone-Butanol-Ethanol fermentation using various extractants. *Energy Conversion and Management* **156**, 288-300 (2018).
