# Supplementary material for: Renewable fatty acid ester production in Clostridium
Source: Nat Commun. 2021 Jul 16;12:4368. doi: 10.1038/s41467-021-24038-3 (PMC8285483; doi:10.1038/s41467-021-24038-3)
Supplement: Supplementary file 5 — Description of Additional Supplementary Files [file 41467_2021_24038_MOESM5_ESM.pdf]

Description of additional supplementary files

Title: Supplementary Data 1
Description: Carbon balance analysis for the fermentation for ester production.

Title: Supplementary Data 2
Description: Strains and plasmids used in this study.

Title: Supplementary Data 3
Description: Primers used in this study.
