# Supplementary material for: Renewable fatty acid ester production in Clostridium
Source: Nat Commun. 2021 Jul 16;12:4368. doi: 10.1038/s41467-021-24038-3 (PMC8285483; doi:10.1038/s41467-021-24038-3)
Supplement: Supplementary file 6 — Reporting Summary [file 41467_2021_24038_MOESM6_ESM.pdf]

nature research

Corresponding author(s): Yi Wang

Last updated by author(s): May 22, 2021

Reporting Summary

Nature Research wishes to improve the reproducibility of the work that we publish. This form provides structure for consistency and transparency in reporting. For further information on Nature Research policies, see our Editorial Policies and the Editorial Policy Checklist.

Statistics

For all statistical analyses, confirm that the following items are present in the figure legend, table legend, main text, or Methods section.

n/a	Confirmed	
☐	☒	The exact sample size (*n*) for each experimental group/condition, given as a discrete number and unit of measurement
☐	☒	A statement on whether measurements were taken from distinct samples or whether the same sample was measured repeatedly
☐	☒	The statistical test(s) used AND whether they are one- or two-sided<br>*Only common tests should be described solely by name; describe more complex techniques in the Methods section.*
☒	☐	A description of all covariates tested
☒	☐	A description of any assumptions or corrections, such as tests of normality and adjustment for multiple comparisons
☐	☒	A full description of the statistical parameters including central tendency (e.g. means) or other basic estimates (e.g. regression coefficient) AND variation (e.g. standard deviation) or associated estimates of uncertainty (e.g. confidence intervals)
☐	☒	For null hypothesis testing, the test statistic (e.g. *F*, *t*, *r*) with confidence intervals, effect sizes, degrees of freedom and *P* value noted<br>*Give P values as exact values whenever suitable.*
☒	☐	For Bayesian analysis, information on the choice of priors and Markov chain Monte Carlo settings
☒	☐	For hierarchical and complex designs, identification of the appropriate level for tests and full reporting of outcomes
☒	☐	Estimates of effect sizes (e.g. Cohen's *d*, Pearson's *r*), indicating how they were calculated

Our web collection on statistics for biologists contains articles on many of the points above.

Software and code

Policy information about availability of computer code

Data collection	Zeiss EM10 transmission electron microscope controlled by MaxIm DL5 software (Cyanogen Imaging, Ottawa, ON, Canada) was used to acquire images. Olympus BX53F Upright Microscope equipped with an Olympus DP73 Camera (Olympus Corporation, Shinjuku-ku, Japan) was used to acquire phase contrast images. High-performance liquid chromatography (Agilent Technologies 1260 Infinity series, Santa Clara, CA) with a refractive index Detector (RID), equipped with an Aminex HPX-87H column (Bio-Rad Laboratories, Hercules, CA) was used for glucose, xylose, butanol, ethanol, lactate, acetate, butyrate and isopropanol measurement. Gas chromatography-mass spectrometry (GC-MS, Agilent Technologies 6890N, Santa Clara, CA) equipped with an HP-5 column (60m×0.25 mm, 0.25 mm film thickness) was used for ethyl acetate, ethyl butyrate, butyl acetate and butyl butyrate measurement. SuperPro Designer software (v11.0, Intelligen Inc., NJ) was used for conducting the process simulation and Microsoft excel spreadsheet (v16.38, Microsoft Corporation, WA) was used for economic and sensitivity analyses.
Data analysis	TEM mage acquisition was performed and analyzed with MaxIm DL5 software (Cyanogen Imaging, Ottawa, ON, Canada). Fermentation data were analyzed using Microsoft Excel 2016 (Microsoft Corporation, WA). Origin 2016 (OriginLab Corporation, MA) and Python 3.7 (Python Software Foundation, DE) were used for statistical analysis.

For manuscripts utilizing custom algorithms or software that are central to the research but not yet described in published literature, software must be made available to editors and reviewers. We strongly encourage code deposition in a community repository (e.g. GitHub). See the Nature Research guidelines for submitting code & software for further information.

Data

Policy information about availability of data

All manuscripts must include a data availability statement. This statement should provide the following information, where applicable:

- Accession codes, unique identifiers, or web links for publicly available datasets
- A list of figures that have associated raw data
- A description of any restrictions on data availability

Data supporting the findings of this work are available within the paper and its Supplementary Information files. A reporting summary for this article is available as a Supplementary Information file. The datasets and other materials generated and analyzed during the current study are available from the corresponding authors upon request. Source data are provided with this paper.

Field-specific reporting

Please select the one below that is the best fit for your research. If you are not sure, read the appropriate sections before making your selection.

☒ Life sciences ☐ Behavioural & social sciences ☐ Ecological, evolutionary & environmental sciences

For a reference copy of the document with all sections, see nature.com/documents/nr-reporting-summary-flat.pdf

Life sciences study design

All studies must disclose on these points even when the disclosure is negative.

Sample size	No sample-size calculation was performed. Sample size was determined to be adequate for data analysis purpose. This study is single strain engineering, and thus sample size is not applicable.
Data exclusions	No data were excluded from the analysis.
Replication	Biological triplicates were used to confirm the reproducibility of the serum bottle fermentations. All attempts showed in this study were successful.
Randomization	Not applicable. We carried out metabolic engineering on a specific strain and used the engineered strains for fermentations, and thus randomization is not applicable in this study.
Blinding	Not applicable. We rationally designed the experiment to achieve our goal, and thus blinding is not applicable.

Reporting for specific materials, systems and methods

We require information from authors about some types of materials, experimental systems and methods used in many studies. Here, indicate whether each material, system or method listed is relevant to your study. If you are not sure if a list item applies to your research, read the appropriate section before selecting a response.

Materials & experimental systems

n/a	Involved in the study	
☒	☐	Antibodies
☒	☐	Eukaryotic cell lines
☒	☐	Palaeontology and archaeology
☒	☐	Animals and other organisms
☒	☐	Human research participants
☒	☐	Clinical data
☒	☐	Dual use research of concern

Methods

n/a	Involved in the study	
☒	☐	ChIP-seq
☒	☐	Flow cytometry
☒	☐	MRI-based neuroimaging
